# Tea Bags for Fmoc Solid-Phase Peptide Synthesis: An Example of Circular Economy

**DOI:** 10.3390/molecules26165035

**Published:** 2021-08-19

**Authors:** Fanny Guzmán, Adriana Gauna, Tanya Roman, Omar Luna, Claudio Álvarez, Claudia Pareja-Barrueto, Luis Mercado, Fernando Albericio, Constanza Cárdenas

**Affiliations:** 1 Núcleo Biotecnología Curauma, Pontificia Universidad Católica de Valparaíso, Valparaíso 2373223, Chile; omar.luna.g@gmail.com; 2 Doctorado en Biotecnología, Pontificia Universidad Católica de Valparaíso, Universidad Técnica Federico Santa María, Valparaíso 2373223, Chile; adrygauna@gmail.com (A.G.); tanya.roman.21@gmail.com (T.R.); 3 Networking Centre on Bioengineering, Department of Organic Chemistry and CIBER-BBN, Biomaterials and Nanomedicine, University of Barcelona, 08028 Barcelona, Spain; albericio@ub.edu; 4 Institute for Advanced Chemistry of Catalonia (IQAC-CSIC), Jordi Girona 18-26, 08034 Barcelona, Spain; 5 Laboratorio de Fisiología y Genética Marina (FIGEMA), Centro de Estudios Avanzados en Zonas Áridas (CEAZA), Coquimbo 1781421, Chile; claudio.alvarez@ceaza.cl; 6 Facultad de Ciencias del Mar, Universidad Católica del Norte, Coquimbo 1781421, Chile; 7 Department of Hematology and Oncology, Pontificia Universidad Católica de Chile, Santiago 8320000, Chile; cparejabarrueto@gmail.com; 8 Instituto de Biología, Pontificia Universidad Católica de Valparaíso, Valparaíso 2373223, Chile; luis.mercado@pucv.cl; 9 School of Chemistry, University of KwaZulu-Natal, Durban 4001, South Africa

**Keywords:** simultaneous synthesis, green chemistry, reagents’ recycling

## Abstract

Peptide synthesis is an area with a wide field of application, from biomedicine to nanotechnology, that offers the option of simultaneously synthesizing a large number of sequences for the purpose of preliminary screening, which is a powerful tool. Nevertheless, standard protocols generate large volumes of solvent waste. Here, we present a protocol for the multiple Fmoc solid-phase peptide synthesis in tea bags, where reagent recycling steps are included. Fifty-two peptides with wide amino acid composition and seven to twenty amino acid residues in length were synthesized in less than three weeks. A clustering analysis was performed, grouping the peptides by physicochemical features. Although a relationship between the overall yield and the physicochemical features of the sequences was not established, the process showed good performance despite sequence diversity. The recycling system allowed to reduce N, N-dimethylformamide usage by 25–30% and reduce the deprotection reagent usage by 50%. This protocol has been optimized for the simultaneous synthesis of a large number of peptide sequences. Additionally, a reagent recycling system was included in the procedure, which turns the process into a framework of circular economy, without affecting the quality of the products obtained.

## 1. Introduction

Peptides are an important class of biomolecules with a broad application in several scientific fields: from biochemical probes to diagnostic kits, from drugs to delivery systems, and from affinity chromatography probes to new materials. This extensive work with peptides would not have been possible without the existence of a robust synthetic method, the solid-phase peptide synthesis (SPPS), developed by R. Bruce Merrifield in the early 1960s [1].

This method is based on the use of a polymeric and solid protecting group for the C-terminal carboxylic acid, and the elongation of the peptide chain is carried out by successive and repetitive steps involving removal of the α-amino protecting group, the incorporation of the protected amino acids of the sequence, and washings between these two previous steps.

The simple idea associated with this method of carrying out all chemistry on a polymer, mainly microporous polystyrene, has added an enormous versatility to the synthetic process and fueled innovative technologies. Thus, to the incredulity of a large number of organic chemist colleagues, the SPPS has been automated. Nowadays, there are several automatic synthesizers that allow the preparation of large peptides with a more than decent purity in a few hours [2]. In the late 1980s, the SPPS was the basis for the development of the combinatorial chemistry, based on the preparation of peptide mixtures. Independent efforts from the groups of Houghten [3] and Lam [4] allowed the preparation of millions of peptides for biological screening, being quite useful in the early phases of drug discovery. While Houghten proposed the screening in solution of mixtures of peptides, Lam preferred the screening of mixtures of beads, containing the peptides, on the basis of “one bead, one peptide/compound”. This latter technology adds the concept of “miniaturization” to the SPPS, because each bead could be considered a nanoreactor, which allows the synthesis of tiny amounts of peptides, just for screening. With the same idea of miniaturization, Geysen [5] and Frank [6] developed strategies for the parallel synthesis of a large number of peptides in polyethylene rods and in membranes, respectively.

All these technologies have their roots in the seminal work of Houghten, who was the first to realize the potential of applying parallelization to the SPPS and using microreactors in the so-called “tea bag” strategy for the concurrent synthesis of a large number of peptides [7]. Thus, the polystyrene resin is located in small polypropylene and sealed packets, which constitute the microreactors. Very interestingly, this strategy allows to carry out not only parallel synthetic steps, but also simultaneous synthetic steps to the ones that do not imply diversity (e.g., washing and deprotection). Furthermore, the “tea bag” technology adds a novelty in relation to the spatial arrangement between the microreactors (“tea bag”) and the reagents and solvents. In most of the organic chemical reactions, the reagents and the solvents are added to the reactors. In the “tea bag” technology, it is the opposite, the microreactors are added to the reagents (for reaction) and to the solvents (for washings). This is key for the simultaneity of a large number of steps, because several reactors could be added to the same reagent (activated protected amino acid or reagent used to remove the protecting group) for reaction, or to the same solvent (for washing). An additional advantage of this strategy is that it allows the preparation of tens of mg of peptides, which could be used for more than one biological assay.

As SPPS continues to gain interest within the research community and pharmaceutical companies, concerns regarding manufacturing costs and large amounts of highly hazardous reagents and solvents also grow [8,9]. As a consequence, environmental sustainability in the production of peptides is a priority. In this way, our groups have been working on the “tea bag” for many years, using the fluorenylmethoxycarbonyl (Fmoc)/tert-butyl (tBu) protection scheme in contrast to Houghten, who utilized the less friendly tert-butyloxycarbonyl (Boc)/benzyl (Bzl) scheme. In addition, we have analyzed new Fmoc removal reagents with advantages regarding toxicity and handling [10]. Thus, during that time, we have optimized all the parameters of this strategy, and herein, we describe a comprehensive protocol detailing all of the steps.

Considering the large volume of polar aprotic solvents, such as N,N-dimethylformamide (DMF), which constitutes the vast majority of the waste generated during peptide synthesis [8], the strategy presented in this report can be framed within the efforts of greening SPPS methodology and circular economy, mostly in reference to the solvents used for the washing steps, which are common for all types of microreactors and can be easily reused for different washings, and the time and the person power needed for the preparation of a large number of peptides. The use of this strategy clearly reduces both parameters, waste generation and time and person power, fulfilling the green concept.

**Figure 1:**
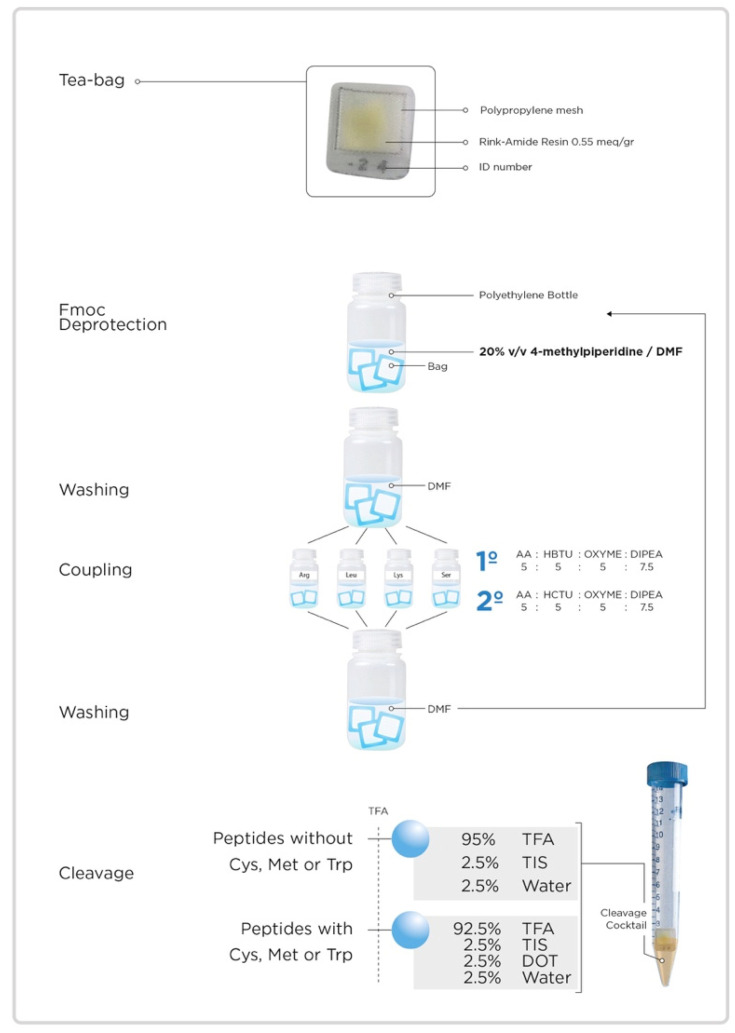
Schematic representation of the tea bag strategy for simultaneous solid-phase peptide synthesis.

Briefly, this protocol for the simultaneous synthesis of tens to hundreds of peptides involves the following steps. Each peptide is synthesized independently in a “tea bag” microreactor, where the common steps (washing and removal of the Fmoc group) are carried out using the same solution from a polyethylene bottle. On the other hand, the coupling of the amino acids is carried out simultaneously for each amino acid in a different polyethylene bottle (one for each amino acid, e.g., 20 if only proteinogenic amino acids are used). Final global deprotection and cleavage is carried out independently for each peptide, allowing an optimization through the use of different cocktails of reagents. Figure 1 illustrates the basic features of the strategy.

## 2. Materials and Methods

### 2.1. Important Issues (Process Generalities)

#### 2.1.1. Quality Practices in Basic Biomedical Research (QPBR)

As a research laboratory, the QPBR were followed according to the general rules of the World Health Organization [11], adapted to the specific requirements as a laboratory for organic synthesis. In addition to the facilities, equipment, reagents and safety standards, the laboratory must have the documentation corresponding to the Standard Operating Procedures (SOPs) for the different operations, and all the syntheses must be carried out by three people, a supervisor and two performers, in order to guarantee compliance with the standards.

#### 2.1.2. Security Measures

The handling of chemical reagents must be carried out with the recommended measures in accordance with its safety data sheet. The laboratory must have an extraction cabinet for solvent handling. The use of nitrile gloves, safety goggles and lab coat are mandatory.

#### 2.1.3. Waste Disposal

All solvents were disposed in appropriate containers and managed by a specialized company. N,N-dimethylformamide (DMF) waste should be separated from those of dichloromethane (DCM) (halogenated compounds) and from those containing ether.

#### 2.1.4. Reaction Flasks and Labeling

All reaction bottles must be resistant to the solvents used in the synthetic process. Therefore, it is recommended to use Nalgene™ containers and VWR^®^ tubes (Merck KGaA, Darmstadt, Germany). Likewise, labeling in all the steps must be carried out in such a way to prevent its fading away when handling the containers, and this is especially critical in the final step of cleavage when the peptides are handled individually.

### 2.2. Reagents and Materials for Synthesis

- Polypropylene mesh sheets (catalog number F7221030-72. www.lumiteinc.com (accessed on 25 June 2021)).
- Resin: Fmoc-Rink Amide AM resin (0.55 meq/g).
- Fmoc-Amino Acids: Fmoc-L-Ala-OH. Fmoc-L-Arg(Pbf)-OH. Fmoc-L-Asn(Trt)-OH. Fmoc-L-Asp(OtBu)-OH. Fmoc-L-Cys(Trt)-OH. Fmoc-L-Gln(Trt)-OH. Fmoc-L-Glu(tBu)-OH. Fmoc-L-Gly-OH. Fmoc-L-His(Trt)-OH. Fmoc-L-Ile-OH. Fmoc-L-Leu-OH. Fmoc-L-Lys(Boc)-OH. Fmoc-Met-OH. Fmoc-L-Phe-OH. Fmoc-L-Pro-OH. Fmoc-L-Ser(tBu)-OH. Fmoc-L-Thr(tBu)-OH. Fmoc-L-Trp(Boc)-OH. Fmoc-L-Tyr(tBu)-OH. Fmoc-L-Val-OH.
- Activating reagents: N-[(1*H*-benzotriazol-1-yl)-(dimethylamino)methylene]-N-methylmethanaminium hexafluorophosphate N-oxide (HBTU). N-[6-chloro(1*H*-benzotriazol-1-yl)-(dimethylamino)methylene]-N-methylmethanaminium hexafluorophosphate N-oxide (HCTU). N,N′-diisopropylcarbodiimide (DIC) and OxymaPure^®^.

Resin, amino acids and activators were purchased from Iris Biotech GmbH (Marktredwitz, Germany). N-ethyldiisopropylamine (DIPEA) and bromophenol blue (BPB) were purchased from Merck KGaA (Darmstadt, Germany).

- Solvents: N,N-dimethylformamide (DMF), 2-propanol (IPA) and dichloromethane (DCM) diethyl ether synthesis grade. Methanol, acetonitrile, ethanol HPLC grade.
- Deprotecting reagent: 4-methylpiperidine (4MP).
- Cleavage Cocktail: trifluoroacetic acid (TFA), triisopropylsilane (TIS), 2.2′-(ethylenedioxy) diethanethiol (DOT); solvents, deprotecting and cleavage reagents were purchased from Merck KGaA (Darmstadt, Germany).

All the abbreviations used can be found in Abbreviation.

### 2.3. Required Documentation

To comply with QPBR, the process of SPPS was monitored with the appropriate documentation, that includes: the working document (described in Section 2.4.2), the formats for calculating the reagent concentration, the formats for the control of reagent preparation, and standard operating procedures developed for each step of the process (reagent preparation, cleavage, purification, HPLC analysis, mass spectrometry analysis, etc.). An example of the work document is presented in the Supplementary Material.

### 2.4. Simultaneous Multiple-Peptide Synthesis “Mise en Place”

#### 2.4.1. Preparation of Tea Bags

**Figure 2:**
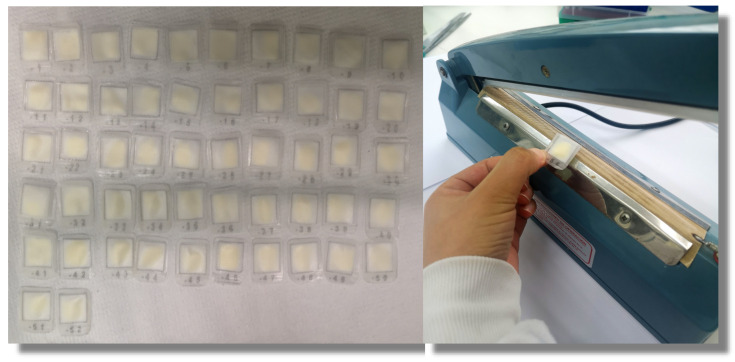
Preparation and sealing of the tea bags.

Squares of 2.5 × 2.5 cm were printed from a file (excel for example) in the polypropylene mesh and pre-sealed and cut, then filled with approximately 40 mg of resin (Figure 2) (loading 0.55 mmol/g and 100–200 mesh). These bags are suitable for up to 20 residue peptides. Note 1: The filling of the bags was carried out by weighing the resin in an Eppendorf tube, labeling the tube according to the resin quantity, and then measuring the resin according to the label, instead of weighing each time, to speed up the filling process, which is especially important when the synthesis includes a large number of peptides.

#### 2.4.2. Working Document

Our lab has a program developed on a server through a PHP script [12] that allows to create a working document for each synthesis. The input data are the sequences of the peptides to be synthesized, the family, defined by the name of the protein from which they come with the position within it, and the respective bag numbers. In the output data, the sequences are distributed, according to the number of bags, in the different coupling cycles, and their length and molecular weight are also calculated. The document also contains the data to calculate the preparation of the stock solutions every 10 couplings. The generated document was the basis for the preparation of the solutions and the implementation of the synthesis (work document in Supplementary material). The program is available online at http://www.acuapeptide.cl/programas.html (accessed on 1 August 2021). In this step, the information are uploaded in the program. For this report, 52 peptides with diverse features were synthesized (see Results section and working document in Supplementary material).

#### 2.4.3. Stock Solutions

To expedite the synthesis process, stock solutions of the reagents were prepared according to a spreadsheet, including the reagents to be used as shown in Table 1. In this table, the amount of reagent per bag per coupling reaction was calculated as follows:

(1) $$mg\;of\;solid\;reagent=Resin\;Loading\;\left(\frac{mmol}{g}\right)\times g\;resin\times MW\;\left(\frac{mg}{mmol}\right)\times fold\;excess$$

(2) $$\mu L\;of\;liquid\;reagent=\frac{Resin\;Loading\;\left(\frac{mmol}{g}\right)\times g\;resin\times MW\;\left(\frac{mg}{mmol}\right)\times fold\;excess}{Density\;\left(\frac{mg}{\mu L}\right)}$$

##### Stock Amino Acid Solutions

Note 1: Due to their susceptibility to air oxidation, Fmoc-L-Cys(Trt)-OH, Fmoc-L-His (Trt)-OH, Fmoc-Met-OH, and Fmoc-L-Trp(Boc)-OH are always prepared just before use for each coupling, using 5 meq/tea bag (Table 1).

The other amino acid solutions are prepared weekly by weighing the amount indicated in Table 1 for 10-fold excess. Stock solutions are prepared for 10 coupling cycles, which is the expected weekly task work, with 2 coupling cycles per day. These data are in the working document, and 1 mL per bag for each amino acid is prepared. Stock solutions are stored refrigerated at 4 °C in properly labeled Nalgene bottles.

##### Stock Activator Solutions (HBTU, HCTU)

Activator solutions are prepared with OxymaPure, by weighing the amount indicated in Table 1 for 10 meq, multiplied by 100, of each activator and OxymaPure as well, and dissolved in 100 mL of DMF. This is considered for 2–4 coupling cycles. Several dry stocks can be previously weighed and then dissolved prior to use. Dry stocks and solutions are stored refrigerated at 4 °C in properly labeled Nalgene bottles. It is possible to use the activators that are available, among the great diversity that exists [13]. The coupling reaction with DIC/Oxyma exhibits neutral pH conditions, whereas phosphonium or uranium salts together with DIPEA show basic conditions. Bromophenol blue as a coupling indicator requires basic conditions for its development, and this makes DIC unsuitable as an activator in this context.

##### Deprotection Reagent (4MP)

20% (*v*/*v*) 4-methylpiperidine (4MP) solution is prepared daily by mixing 40 mL of the deprotection reagent with 160 mL of DMF. Solutions are kept at room temperature (RT) in properly labeled WMR tubes. Solution is meant to be used within the workday considering two deprotection/coupling cycles per day.

##### Coupling Indicator (BMP)

0.1% (*w*/*v*) bromophenol blue solution is prepared by weighing 0.5 g of bromophenol blue and dissolving it in 0.5 L of DMF. Reagent solutions are stable when kept at 4 °C for up to two weeks; however, we did not use solutions older than one week.

### 2.5. Synthesis Protocol

#### 2.5.1. Resin Swelling

Resin in the bags is swelled in a reaction vessel (polyethylene bottle), with DCM for 30 min and then with DMF for 30 min. Solvents are then discarded. This step, in addition to swelling the resin, is necessary to test the sealing of the bags, verifying that there is no loss of resin, as well as its correct labeling, verifying that the tea bag numbers are still clearly visible after the process.

#### 2.5.2. Initial Deprotection

**Figure 3:**
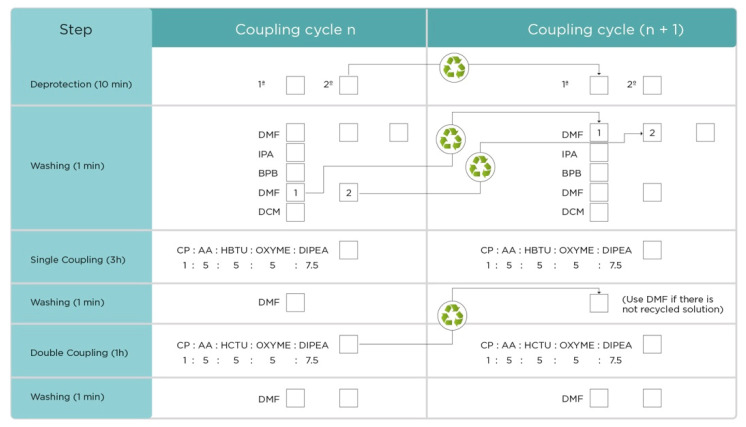
Schematic diagram of the coupling steps and reagents recycling in the cycle.

In a reaction vessel for all bags, the first resin-linked Fmoc removal step is carried out adding enough deprotection reagent to cover the tea bags (4MP stock solution) and agitating mildly at RT for 10 min. This step is repeated once. Solutions are discarded in each step. Resin is then washed thrice with DMF for 1 min, once with IPA for 1 min, once with bromophenol blue for 2 min, and twice with DMF for 1 min (the solutions from the last two washings are recycled according to Figure 3, for the next deprotection step after the first coupling). After this step, the process continues from single coupling step.

#### 2.5.3. Coupling Cycle

##### Deprotection

**Figure 4:**
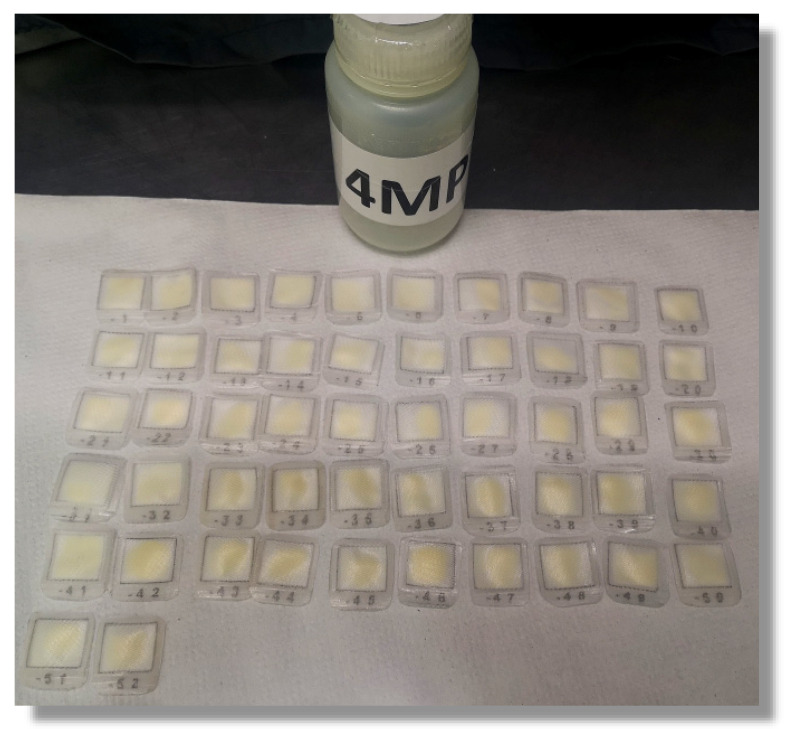
Fmoc deprotection step before coupling. All the tea bags are put in a single container.

**Figure 5:**
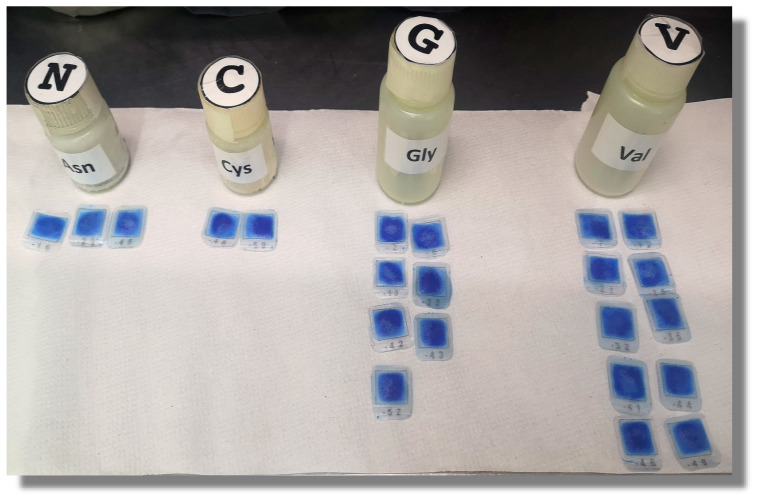
Distribution of the tea bags according to the amino acid to be coupled, after washing with bromophenol blue.

Note 2: All deprotection cycles are carried out according to a recycling scheme (Figure 3). Each deprotection step is performed twice for 10 min each, and the second deprotection solution is recycled to be used in the next cycle. Resin is then washed thrice with DMF for 1 min, once with IPA for 1 min, once with bromophenol blue for 2 min, and twice with DMF for 1 min (the solutions from the last two washings are recycled according to Figure 3, for the next deprotection step). This step is performed at the beginning of the coupling cycle, which is repeated as many times as needed while amino acids are in the peptide to be synthesized (Figure 4). Resin should be blue after the deprotection due to the effect of bromophenol blue (see colorimetric test and Figure 5).

##### Single Coupling

A coupling mix is prepared in labeled Nalgene bottles mixing 0.5 mL/bag of both amino acid and HBTU/OxymaPure activator stock solutions. Tea bags are distributed in groups according to the amino acids in the sequence, starting with the C-terminal amino acid of each peptide. One performer reads the bag ID from the working document while the other indicates to which amino acid this bag ID corresponds. Bags are placed in front of the bottle containing their current coupling mix (Figure 5). A double check is carried out before the bags are placed in their corresponding coupling mix. DIPEA is added to the coupling mix according to the number of bags in each bottle (29 µL per bag, Table 1). Whenever possible, coupling is carried out at 45 °C in quiescent incubation for 10 min, followed by agitation at 25 °C until completing 3 h of total coupling time. Solution is discarded and bags are washed with DMF for 1 min once, into the individual bottles. Solvent is discarded.

##### Double Coupling

A second coupling mix is prepared, in the same way as performed for the single coupling, but changing the activator to HCTU and adding it to the corresponding labeled bottles containing the bags. DIPEA is added, and this time a coupling time of 1 h is used. This solution is saved to wash the bags after single coupling of the respective amino acid before adding the double coupling mix, in the next coupling cycle (Figure 3).

##### Colorimetric Test 

Bromophenol blue binds to the N-terminal free amines in the growing chains at neutral and basic pH values (side-chain amines, such as Lys, remain protected throughout the synthesis), thus a blue resin color reflects that the Fmoc protecting group has been removed and blue/green shades of color after single or double coupling steps indicate an incomplete coupling [14,15]. When the color indicate an incomplete coupling a third coupling is then necessary, changing the activator. When resin acquires yellow-orange shades, the coupling is considered complete. Note 3: If the bags retain any shade of blue or green, additional couplings should be performed with other activators available. However, going beyond a triple coupling is seldom necessary.

##### Washing

Tea bags that pass the colorimetric test are washed twice with DMF for 1 min. Solvent is discarded. Then, the cycle is repeated until the sequence is completed.

#### 2.5.4. Final Deprotection and Cleavage

After all the required coupling steps are performed, the tea bags undergo a final Fmoc removal step, with the corresponding washings, as previously described in Section 2.5.2, except for bromophenol blue. All solvents used in the final deprotection need to be fresh. Resin is thoroughly dried. The next step will remove the final product from the resin and will simultaneously deprotect the side chains by acidolysis, using TFA in the presence of scavengers.

##### Label and Preparation of the Bags

Tea bags are air-dried and then placed individually in 15 mL Falcon tubes carefully labeled with both the bag ID and peptide ID numbers. A second tube per peptide is also labeled. All the details deemed necessary for the correct identification of the product should be registered in the label. Bags should be pushed carefully to the bottom of the tube. Note 4: the label must resist the solvents and the subsequent freezing process.

##### Preparation of Cleavage Solutions

Note 5: Cys, Met, and Trp residues are prone to oxidation under the acidic conditions of the cleavage procedure. For peptides containing either of these residues, a reducing agent must be added to the cleavage mix. Two cleavage mixtures are prepared considering 1.5 mL/bag. Cleavage Solution 1 is comprised of TFA:TIS:water (95:2.5:2.5). Cleavage Solution 2 is comprised of TFA:TIS:water:DOT (92.5:2.5:2.5:2.5) and is to be used for peptides containing Cys, Met, or Trp residues.

##### Cleavage

1.5 mL of the corresponding cleavage solution is added to each tube, verifying that the tea bag is completely covered. Tubes are carefully sealed with a labeled cap and parafilm and the cleavage reaction is carried out under mild agitation at RT for 3 h. Warning: TFA is corrosive and hazardous to skin, eyes, and mucosae, so contact should always be avoided. Basic safety rules for handling strong acids must be followed. Nitrile gloves, coat, and eye protection must be used, and work under the extraction cabin flow is mandatory. Cleavage reagents must be discarded in acid waste containers.

##### Peptide Precipitation

**Figure 6:**
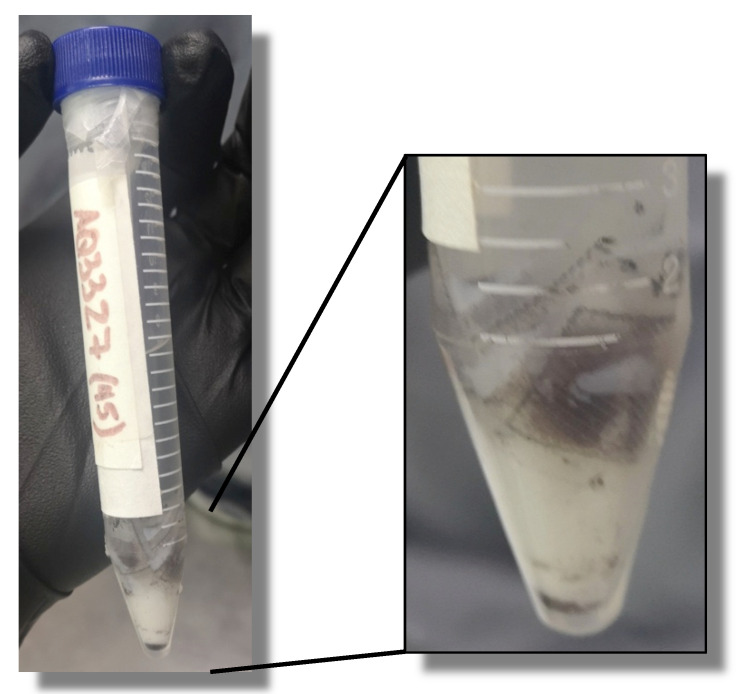
Peptide precipitation with ice-cold diethyl ether.

7 mL of ice-cold diethyl ether (<−20 °C) is added to the tubes, mixing vigorously until a white precipitate is noticeable (Figure 6). Then, the tubes are centrifuged at 3500 rpm for 10 min. The pellet contains most of the cleaved product. Four washings with 3 mL of cold diethyl ether are carried out, mixing vigorously with vortex and pelleting the peptide at 3500 rpm for 3 min. Each time, the supernatant is collected into the second labeled tube and cooled at −20 °C for 10 min if the solubility of the product hinders its initial precipitation and some product would thus later precipitate in the supernatant. This tube is also centrifuged as described, discarding the supernatant. Pellets are air-dried until complete diethyl ether elimination, and then 3 mL of milliQ water is added under stirring to dissolve the peptide, performing this twice and mixing the fractions into the second labeled tube. 3 mL of water is also added to the tube containing the bag if there is peptide remaining. Tubes are frozen at −20 or −80 °C and lyophilized for 48 h.

### 2.6. Characterization

After the synthesis, as a routine process, the characterization should be carried out by high-performance liquid chromatography to verify the purity and by mass spectrometry to verify the identity of the product.

#### 2.6.1. Reversed Phase High-Performance Liquid Chromatography (HPLC)

Peptides are analyzed by RP-HPLC, acquiring at 214 nm, which is the absorption wavelength of the peptide bond. However, when aromatic amino acids are present, absorption increases, so the amount of peptide for this analysis depends on the sequence. A range between 2 and 20 µg is adequate for injection, with 10 µg being a good standard load to obtain a quality signal for many peptides. Stock samples of 1 µg/µL of peptide are prepared. Analysis was carried out in HPLC system equipment comprised of an Autosampler AU-2055 Plus Quaternary Gradient Pump PU-2089 Plus and UV-2075 Plus UV-Visible Detector (JASCO Corp., Tokyo, Japan) using a XBridge™ BEH C18 column (100 × 4.6 mm, 3.5 µm) (Waters Corp., Milford, MA, USA). Peptides were analyzed with a 0–70% acetonitrile gradient in 10 min. Note 6: The two solutions used were: solution A (milliQ water + 0.05% (*v*/*v*) TFA) and solution B (acetonitrile + 0.05% (*v*/*v*) TFA). Samples were prepared by adding 10 μL of stock (1 µg/µL) to 90 μL of milliQ water. Data acquisition and analysis were performed using ChromNAV Chromatography Data System v 2.02.05 Build 4 (JASCO Corp., Tokyo, Japan) software.

#### 2.6.2. Electrospray Ionization-Mass Spectrometry (ESI-MS)

LCMS-2020 ESI-MS (Shimadzu Corp., Kyoto, Japan) equipment was used. 1 µg/µL stock sample solutions were prepared. 10 µL of stock sample solution was loaded and run in positive ion mode at 4.5 kV and 350 °C for 20 min. Data acquisition and analysis were performed using the Lab Solutions v5.42 SP3 software (Shimadzu Corp., Kyoto, Japan).

### 2.7. Purification

Sometimes the synthesis can yield products of up to 90% purity, but if it is necessary to have a product with higher purity, the crude peptides are fractionated using preparative Clean-Up^®^ CEC18153 C-18 extraction columns (UCT, Bristol, PA, USA). Columns were washed twice with 2 mL of methanol and twice with 2 mL of milliQ water. 5–7 mg of the crude peptide was dissolved in milliQ water and loaded onto the column, then the flow-through was collected and reloaded thrice. Elution of the peptide was carried out by adding 0.9–1 mL of successive solutions of water/acetonitrile with concentrations between 0% and 60% (%*v*/*v*), collecting each eluent fraction. Finally, the column was washed twice with 1 mL of acetonitrile and twice with 1 mL of methanol, and the eluent was collected. Acetonitrile was evaporated using a Savant SPD 1010 SpeedVac Concentrator (Thermo Fisher Scientific, Asheville, NC, USA) and the aqueous remnant was frozen at −80 °C to be lyophilized. Fractions were evaluated by HPLC and ESI-MS, to determine the main fraction containing the expected peptide.

## 3. Results

### 3.1. Yield and Purity

As an example, the synthesis of 52 peptides from various proteins related to the immune response is presented. Table 2 shows a summary of the information gathered for the 52 synthesized peptides. Tea bags with 40 mg of resin were used. Theoretical yield (TY) of peptides is calculated according to Equation (3), % of raw peptide yield (RY) is calculated according to Equation (4), and % of pure yield peptide (PY) is calculated considering HPLC integration of the corresponding peak, according to Equation (5).

(3) TY=Loading Capacity∗Molecular Weight

Loading capacity is calculated according to Table 1.

(4) %RY=mg synthesized peptideTY∗100

(5) %PY=HPLC purity∗Raw Yield100  

HPLC purity is obtained for integration of the main peak in the corresponding chromatogram.

### 3.2. Electrospray Ionization-Mass Spectrometry (ESI-MS)

Results of the ESI/MS are shown in Table 2. As can be seen, most of the peptides were correctly identified, with two exceptions: peptide AQ3316, whose mass spectrum did not match the expected peaks, and peptide AQ3317, whose mass analysis showed the deletion of an arginine residue.

HPLC chromatograms and can be seen in Supplementary material Table S1.

Some of the yields are above 100, which is mainly due to two factors: the filling of the bags (Note 1 in Section 2.4.1) and the average value of the resin substitution, and these factors can produce a ±20% variation.

Additionally, a set of descriptors for each peptide was calculated (Supplementary material Table S2) to characterize them in order to establish a trend between the physicochemical features of the peptides and the yield of the synthesis. Characterization of each peptide was carried out using the Peptide package [16] implemented in RStudio [17].

Peptide sequences were characterized by the descriptors in Table S2 and clustered after a principal component analysis (PCA) to obtain the maximum variance of the dataset. The descriptors included properties related to size, polarity, hydrophobicity, and some electronic properties that can be considered to have some influence in the peptide synthesis process (Supplementary material Table S3). Four PC were selected, accounting for 96% of the variance (Supplementary material Table S4).

**Figure 7:**
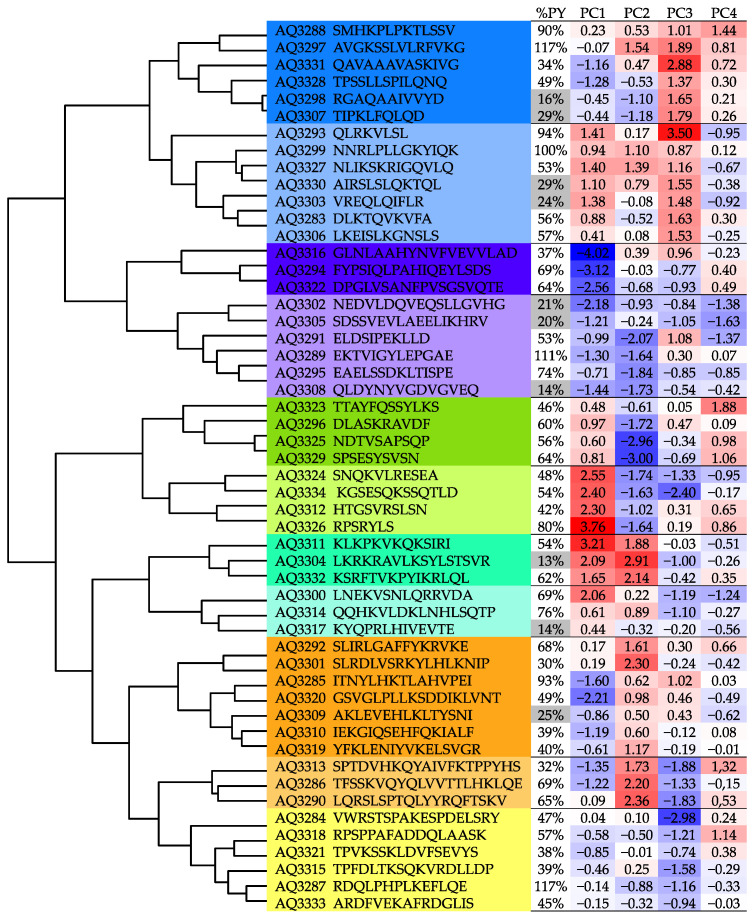
Clustering analysis of the 52 synthesized peptides. Clusters are identified by colors and the principal components are in a scale of red to blue from higher to lower values. Pure yields are indicated in the first column, with the values below 29% highlighted in grey.

Each cluster can be associated mainly with one or two principal components, and likewise with the original variables according to the factor loadings that correlate these variables with each PC (Supplementary material Table S5), as can be seen in Figure 7 and Table 3.

As can be seen, the synthesized peptides show a great diversity according to the cluster analysis, with at least eleven differentiated clusters.

However, despite this diversity, the synthesis yield, in terms of pure peptide yield, shows that more than 80% of the peptides had a yield above 30%, with only 19% of them with yields between 13% and 29% (Supplementary material Figure S1).

Although it is difficult to define what are the characteristics that determine the performance of a peptide when synthesized, there are trends that can influence it. For example, hydrophobic sequences tend to present low synthesis yields; in this case, clusters 3 and 4 are conformed by hydrophobic sequences, and the synthesis presented high yields. Additionally, the longest sequences, in cluster 10, have a high yield. These facts reinforce the advantages of the tea bag synthesis for its use in a wide range of applications.

### 3.3. Recycling and Reagent Usage

In order to reduce the amounts of solvents and reagents, the steps in this protocol where it is possible to incorporate a recycling scheme have been indicated (Figure 3). In this protocol, the solution employed for the second step in the deprotection stage is recycled in the first step for the next cycle (deprotection step). By doing so, the use of the deprotection reagent was reduced by 50%. Previously, our group tried to carry out the Fmoc removal step using a fresh solution of 10% *v*/*v* of the deprotection reagent (halving the usually recommended concentration); however, the purity and yield of most of the products in that synthesis were severely reduced (data not shown). The recycling scheme for deprotection thus allows us to maintain a 50% reduction in reagent usage without reducing the overall quality of the products.

Additionally, the recycling scheme was extended to the washing steps after the deprotection reaction, in which the two final DMF washings (single coupling step) were used in the first two washing steps for the next cycle. In this way, the entire recycling system allows to reduce DMF usage by 25–30%, and deprotection reagent usage by 50%, resulting in considerable savings in terms of costs and waste generation. Table 4 shows a comparison of solvent consumption for the syntheses carried out in this report, together with syntheses in tea bags without recycling, and a synthesis in individual reactors, based on 52 peptides at an average of 15 couplings.

## 4. Conclusions

A protocol for the simultaneous synthesis of many peptides has been presented, using the Fmoc/tBu tea bag strategy, based on Houghten’s protocol initially proposed for the Boc/Bzl approach [17].

The coupling cycle was standardized using an activator for single coupling (HBTU) and another for double coupling (HCTU), which can be interchangeable. If an additional coupling is required, any other available activator can be used.

In general, the protocol detailed in this work permitted to obtain 52 different peptide sequences with different length, hydrophilicity and sequence difficulties, with a high level of purity, reducing at the same time the reagents’ usage and waste generation within the framework of the new trends of making the peptide synthesis more environmentally friendly. Two out of these fifty-two peptide sequences were not properly synthesized, which is within the margin of experimental error of the procedure. Actually, these two sequences are considered hard to synthesize and therefore more prone to error.

This protocol allowed synthesizing from tens to hundreds of peptides in a short time and with a good yield to be used in different research projects. The amount of peptide can be doubled or even tripled, if necessary, by simply including more than one bag for the same sequence. This is an additional advantage of this method: each peptide can be obtained in different amounts as required.

This protocol has demonstrated to be quite useful when a large number of sequences need to be synthesized. Although it can be used for the preparation of totally unrelated sequences, it is particularly convenient in the case of protein epitope mapping [18,19], in the case of Ala scan for the identification of key residues in a peptide [20,21], to test different strategies or reagents [10], in the synthesis of antigen epitopes for use in diagnosis (antibody generation) [22,23], in the search of new compounds [24,25], and in the development of peptide-based vaccines, among other applications.

It is important to highlight that the strategy presented herein is an excellent approximation to the green circular economy concept [26], which is based on the minimization of reagents (parallel synthesis using the same reagent solutions for completely different peptide syntheses) and waste reclaiming (second deprotection solution in one cycle is used as the first one in the next cycle, and the last DMF washings of a cycle are used as the first washings in the next cycle). Importantly, the quality of the final product was not affected and therefore the principle of reusing the deprotection and the washings solutions can be envisaged as a sound strategy to be transferred to the batch protocol in the large-scale synthesis of peptides using SPPS. Further studies in our laboratory are focused on including more recycling processes.

## Figures and Tables

**Table 1:** Spreadsheet to calculate the quantities for reagents’ stock solutions.

Amino Acid	Reagent	MW (g/mol)	1 meq (mg)	5-Fold Excess (mg)	10-Fold Excess (mg)
ALA	A	311.3	6.8	34.2	68.5
ARG	R (Pbf)	648.8	14.3	71.4	142.7
ASN	N (Trt)	596.7	13.1	65.6	131.3
ASP	D (tBu)	411.5	9.1	45.3	90.5
CYS	C (Trt)	585.7	12.9	64.4	128.9
PHE	F	387.4	8.5	42.6	85.2
GLY	G	297.3	6.5	32.7	65.4
GLU	E (tBu)	443.5	9.8	48.8	97.6
GLN	Q (Trt)	610.7	13.4	67.2	134.4
HIS	H (Trt)	619.7	13.6	68.2	136.3
ILE	I	353.4	7.8	38.9	77.7
LEU	L	353.4	7.8	38.9	77.7
LYS	K (Boc)	468.5	10.3	51.5	103.1
MET	M	371.5	8.2	40.9	81.7
PRO	P	337.4	7.4	37.1	74.2
SER	S (tBu)	383.4	8.4	42.2	84.3
TYR	Y (tBu)	459.6	10.1	50.6	101.1
THR	T (tBu)	397.5	8.7	43.7	87.5
TRP	W (Boc)	526.6	11.6	57.9	115.9
VAL	V	339.4	7.5	37.3	74.7
	HBTU	379.3	8.3	41.7	83.4
	HCTU	413.69	9.1	45.5	91.0
	TBTU	321.1	7.1	35.3	70.6
	TCTU	355.5	7.8	39.1	78.2
	OxymaPure	142.11	3.1	15.6	31.26
	DIPEA	129	5.7	28.7	57.37
	DIC	126.2	3.4	16.9	33.9
Loading Capacity (LC)		0.022	LC=RS×Resin (g)		
Resin Subst. (RS)		0.55	meq=LC×MW (Solid reagents)		
Resin, g		0.04	meq=LC×MW×1.5density (DIPEA)		
			meq=LC×MWdensity (Liquid reagents)		

DIPEA base is always used in 1.5 eq. with respect to all other reagents in the coupling mix. Stock solutions are detailed in the following list.

**Table 2:** Synthesized peptides and their main features.

Label	Bag	Sequence	#Residues	MW	Mass Spectrometry Analysis						HLPC Purity	Theoretical Yield (mg)	Experimental Yield (mg)	%RY	%PY
						M1+	M2+	M3+	M4+	M5+					
AQ3283	1	DLKTQVKVFA	10	1147.6	ok	1148.6	575.3	383.9	288.2	230.7	77.4	27.5	19.9	72	56
AQ3284	2	VWRSTSPAKESPDELSRY	18	2107.3	ok	2108.3	1055.2	703.8	528.1	422.7	56.4	50.6	42.2	83	47
AQ3285	3	ITNYLHKTLAHVPEI	15	1748.1	ok	1749.1	875.55	584.0	438.3	350.8	96.6	42.0	40.4	96	93
AQ3286	4	TFSSKVQYQLVVTTLHKLQE	20	2348.7	ok	2349.7	1175.9	784.2	588.4	470.9	67.4	56.4	57.5	102	69
AQ3287	5	RDQLPHPLKEFLQE	14	1749.0	ok	1750.0	876.0	584.3	438.5	351,0	95.2	42.0	51.4	122	117
AQ3288	6	SMHKPLPKTLSSV	13	1423.7	ok	1424.7	713.35	475.9	357.2	285.9	94.9	34.2	32.6	95	90
AQ3289	7	EKTVIGYLEPGAE	13	1404.6	ok	1405.6	703.8	469.5	352.4	282.1	83.4	33.7	45.1	134	111
AQ3290	8	LQRSLSPTQLYYRQFTSKV	19	2314.7	ok	2315.7	1158.9	772.9	579.9	464.1	73.8	55.6	49.1	88	65
AQ3291	9	ELDSIPEKLLD	11	1270.4	ok	1271.4	636.7	424.8	318.9	255.3	48.1	30.5	33.5	110	53
AQ3292	10	SLIRLGAFFYKRVKE	15	1826.2	ok	1827.2	914.6	610.1	457.8	366.4	62.2	43.8	47.8	109	68
AQ3293	11	QLRKVLSL	8	955.2	ok	956.2	479.1	319.7	240.1	192.2	58.8	22.9	18.4	80	47
AQ3294	12	FYPSIQLPAHIQEYLSDS	18	2107.3	ok	2108.3	1055.2	703.8	528.1	422.8	77.2	50.6	45.3	90	69
AQ3295	13	EAELSSDKLTISPE	14	1517.6	ok	1518.6	760.3	507.2	380.7	304.7	85.3	36.4	31.5	87	74
AQ3296	14	DLASKRAVDF	10	1120.3	ok	1121.3	561.7	374.8	281.3	225.3	49.9	26.9	32.1	120	60
AQ3297	15	AVGKSSLVLRFVKG	14	1459.8	ok	1460.8	731.4	487.9	366.2	293.2	97.4	35.0	41.9	120	117
AQ3298	16	RGAQAAIVVYD	11	1161.3	ok	1162.3	582.2	388.4	291.6	233.5	19.1	27.9	23.5	84	16
AQ3299	17	NNRLPLLGKYIQK	13	1555.9	ok	1556.9	779.5	520.0	390.2	312.4	78.8	37.3	47.5	127	100
AQ3300	18	LNEKVSNLQRRVDA	14	1640.8	ok	1641.8	821.9	548.3	411.5	329.4	69.7	39.4	38.7	98	69
AQ3301	19	SLRDLVSRKYLHLKNIP	18	2051.5	ok	2052.5	1027.3	685.2	514.1	411.5	52.5	49.2	28.3	57	30
AQ3302	20	NEDVLDQVEQSLLGVHG	17	1851.0	ok	1852.0	927.0	618.3	464.0	371.4	64.0	44.4	14.3	32	21
AQ3303	21	VREQLQIFLR	10	1300.6	ok	1301.6	651.8	434.9	326.4	261.3	50.1	31.2	14.9	48	24
AQ3304	22	LKRKRAVLKSYLSTSVR	17	2004.4	ok	2005.4	1003.7	669.5	502.4	402.1	38.5	48.1	16.2	34	13
AQ3305	23	SDSSVEVLAEELIKHRV	17	1910.1	ok	1911.1	956.6	638.0	478.8	383.2	72.5	45.8	12.7	28	20
AQ3306	24	LKEISLKGNSLS	12	1287.5	ok	1288.5	645.3	430.5	323.1	258.7	81.9	30.9	21.4	69	57
AQ3307	25	TIPKLFQLQD	10	1204.4	ok	1205.4	603.7	402.8	302.4	242.1	57.3	28.9	14.7	51	29
AQ3308	26	QLDYNYVGDVGVEQ	14	1597.7	ok	1598.7	800.4	533.9	400.7	320.8	30.9	38.3	16.9	44	14
AQ3309	27	AKLEVEHLKLTYSNI	15	1758.0	ok	1759.0	880.5	587.3	440.8	352.8	46.1	37.8	20.6	55	25
AQ3310	28	IEKGIQSEHFQKIALF	16	1887.2	ok	1888.2	945.1	630.4	473.1	378.7	83.4	45.3	21.0	46	39
AQ3311	29	KLKPKVKQKSIRI	13	1565.0	ok	1566.0	784.0	523.0	392.5	314.2	95.0	37.6	21.5	57	54
AQ3312	30	HTGSVRSLSN	10	1056.3	ok	1057.3	529.7	353.4	265.3	212.5	48.3	25.4	22.1	87	42
AQ3313	31	SPTDVHKQYAIVFKTPPYHS	20	2401.8	ok	2402.8	1202.4	801.9	601.7	481.6	78.7	55.6	22.3	40	32
AQ3314	32	QQHKVLDKLNHLSQTP	16	1590.0	ok	1591.0	796.5	531.3	398.8	319.2	97.5	45.2	35.5	78	76
AQ3315	33	TPFDLTKSQKVRDLLDP	17	1972.3	ok	1973.3	987.7	658.8	494.3	395.7	75.5	47.3	24.4	52	39
AQ3316	34	GLNLAAHYNVFVEVVLAD	18	1943.2	No *	1944.2	973.1	649.1	487.1	389.8	87.0	46.6	19.8	42	37
AQ3317	35	KYQPRLHIVEVTE	13	1610.9	No **	1611.9	806.9	538.3	404.0	323.4	37.2	38.7	14.7	38	14
AQ3318	36	RPSPPAFADDQLAASK	16	1669.8	ok	1670.8	836.4	557.9	418.7	335.2	72.8	40.1	31.4	78	57
AQ3319	37	YFKLENIYVKELSVGR	16	1958.5	ok	1959.5	980.8	654,2	490.9	392.9	40.4	47.0	46.9	100	40
AQ3320	38	GSVGLPLLKSDDIKLVNT	18	1869.5	ok	1870.5	936.3	624.5	468.6	375.1	46.8	44.9	46.9	105	49
AQ3321	39	TPVKSSKLDVFSEVYS	16	1786.1	ok	1787.1	894.6	596.7	447.8	358.4	55.1	42.9	29.4	68	38
AQ3322	40	DPGLVSANFPVSGSVQTE	18	1801.8	ok	1802.8	902.4	601.9	451.7	361.6	98.8	43.2	28.2	65	64
AQ3323	41	TTAYFQSSYLKS	12	1394.6	ok	1395.6	698.8	466.2	349.9	280.1	68.2	33.5	22.4	67	46
AQ3324	42	SNQKVLRESEA	11	1259.6	ok	1260.6	631.3	421.2	316.2	253.1	56.4	30.2	25.8	85	48
AQ3325	43	NDTVSAPSQP	10	1014.5	ok	1015.5	508.8	339.5	254.9	204.1	68.7	24.3	19.9	82	56
AQ3326	44	RPSRYLS	7	877.5	ok	878.5	440.3	293.8	220.6	176.7	90.2	21.1	18.8	89	80
AQ3327	45	NLIKSKRIGQVLQ	13	1495.9	ok	1496.9	749.5	500.0	375.2	300.4	92.4	35.9	20.5	57	53
AQ3328	46	TPSSLLSPILQNQ	13	1396.7	ok	1397.7	699.9	466.9	350.4	280.5	71.3	33.5	22.8	68	49
AQ3329	47	SPSESYSVSN	10	1055.4	ok	1056.4	529.2	353.1	265.1	212.3	81.0	25.3	20.0	79	64
AQ3330	48	AIRSLSLQKTQL	12	1356.8	ok	1357.8	679.9	453.6	340.5	272.6	33.4	32.6	28.0	86	29
AQ3331	49	QAVAAAVASKIVG	13	1183.7	ok	1184.7	593.4	395.9	297.2	237.9	45.2	28.4	21.3	75	34
AQ3332	50	KSRFTVKPYIKRLQL	15	1876.1	ok	1877.1	939.5	626.7	470.3	376.4	53.1	45.0	52.6	117	62
AQ3333	51	ARDFVEKAFRDGLIS	15	1722.9	ok	1723.9	862.9	575.6	432.0	345.8	63.3	41.3	29.2	71	45
AQ3334	52	KGSESQKSSQTLD	13	1393.7	ok	1394.7	698.4	465.9	349.7	279.9	54.4	33.4	33.5	100	54

* According to the mass spectroscopy analysis, the peptide was not obtained. ** Mass spectrometry analysis revealed the deletion of one arginine residue.

**Table 3:** PC and original variables associated with each cluster. Clusters are colored with the same color code as in Figure 7.

Cluster	PC	Original Variable
1	PC3	Hidrophobicity/Boman
2	PC3–PC1	Aliphatic index
3	PC1	Hidrophobicity/Aliphatic index
4	PC1–PC2	Charge/pI
5	PC1–PC2	Charge/Aliphatic Index
6	PC1	Hidrophobicity
7	PC1	Charge/Hidrophobicity
8	PC1–PC3	Boman/Hidrophobicity
9	PC1–PC2	pI/Charge
10	PC2–PC3	Size
11	PC3	Aliphatic index

**Table 4:** Comparison of solvent consumption for synthesis with tea bags with and without recycling, and synthesis in an individual reactor.

Item	Tea Bag Recycling	Tea Bag No Recycling	Individual Reactor
	mL	mL	mL
DMF	5000	9000	6240
DCM	1000	2000	2500
IPA	1000	1000	1250
4MP	140	280	750

## Data Availability

Not applicable.

## Supplementary Materials

The following are available online, Table S1: HPLC chromatograms and Mass spectra for the 52 peptides synthesized, Table S2: Descriptors calculated for each peptide sequence, Table S3: Original variables calculated for each peptide sequence, Table S4: Importance of components, Table S5: Absolute values for the PCA factor loadings, Figure S1: Number of peptides grouped by frequency according to the obtained pure yield, and Working_Document.

## Abbreviations

4MP	4-methylpiperidine
ALA	alanine
ARG	arginine
ASN	asparagine
ASP	aspartic acid
Boc	tert-butyloxycarbonyl
BPB	bromophenol blue
Bzl	benzyl
CYS	cysteine
DCM	dichloromethane
DIC	N.N′-diisopropylcarbodiimide
DIPEA	N-ethyldiisopropylamine
DMF	N.N-dimethylformamide
DOT	2.2′-(ethylenedioxy) diethanethiol
ESI-MS	Electrospray Ionization-Mass Spectrometry
Fmoc	fluorenylmethoxycarbonyl
GLN	glutamine
GLU	glutamic acid
GLY	glycine
QPBR	Quality Practices in Basic Biomedical Research
HBTU	N-[(1H-benzotriazol-1-yl)-(dimethylamino)methylene]- N-methylmethanaminium hexafluorophosphate N-oxide
HCTU	N-[6-chloro(1H-benzotriazol-1-yl)-(dimethylamino)methylene]- N-methylmethanaminium hexafluorophosphate N-oxide
HIS	histidine
ILE	isoleucine
IPA	2-propanol
LEU	leucine
LYS	lysine
MET	methionine
PHE	phenylalanine
PRO	proline
PY	peptide yield
RP-HPLC	Reversed Phase-High Performance Liquid Chromatography
RY	raw peptide yield
SER	serine
SOPs	Standard Operating Procedures
SPPS	solid-phase peptide synthesis
tBu	tert-butyl
TFA	trifluoroacetic acid
THR	threonine
TIS	triisopropylsilane
TRP	tryptophan
Trt	trityl
TY	Theoretical yield
TYR	tyrosine
VAL	valine
