# Supplementary material for: Tea Bags for Fmoc Solid-Phase Peptide Synthesis: An Example of Circular Economy
Source: Molecules. 2021 Aug 19;26(16):5035. doi: 10.3390/molecules26165035 (PMC8399505; doi:10.3390/molecules26165035)
Supplement: Supplementary file 1 [file molecules-26-05035-s001.zip › Supplementary_material.pdf]

Supplementary Material

Tea-Bags for Fmoc Solid-Phase Peptide Synthesis. an Example of Circular Economy

Fanny Guzmán*, Adriana Gauna,Tanya Roman, Omar Luna, Claudio Álvarez, Claudia Pareja-Barrueto, Luis Mercado, Fernando Albericio, Constanza Cárdenas*

Table S1. HPLC chromatograms and Mass spectra for the 52 peptides synthesized.

<table>
<tr><th>Label</th><th>RP-HPLC</th><th>ESI-MS</th></tr>
<tr><td>AQ3283</td><td>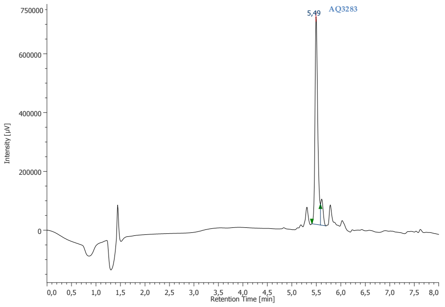
</td><td>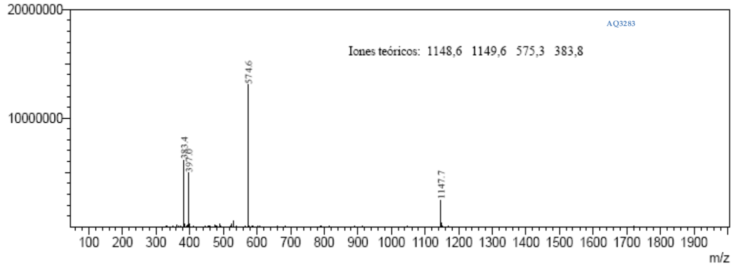
</td></tr>
<tr><td>AQ3284</td><td>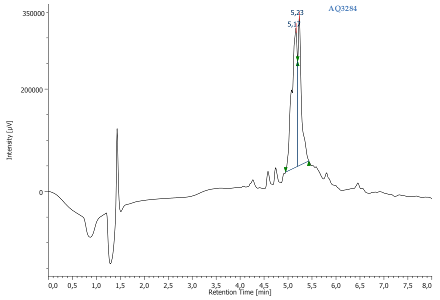
</td><td>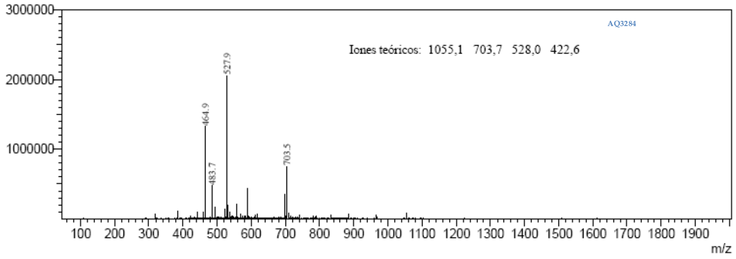
</td></tr>
<tr><td>AQ3285</td><td>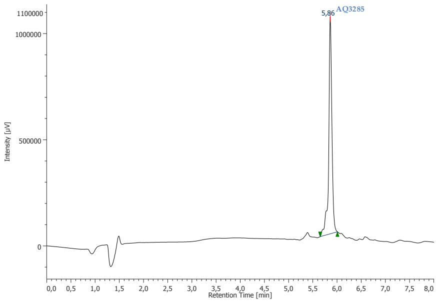
</td><td>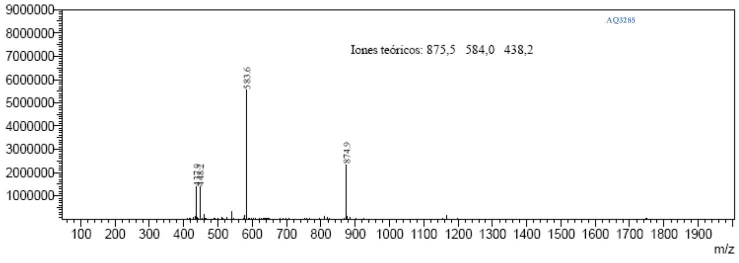
</td></tr>
</table>

AQ3286	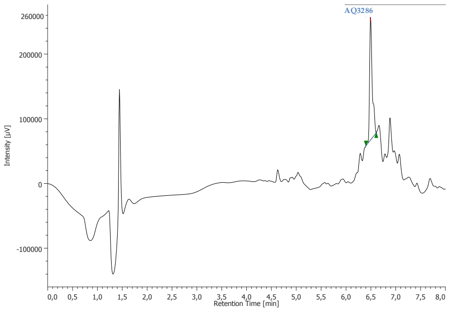	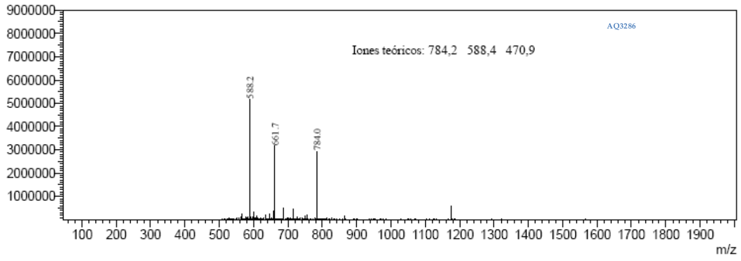
AQ3287	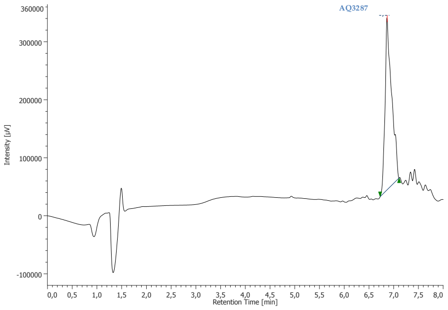	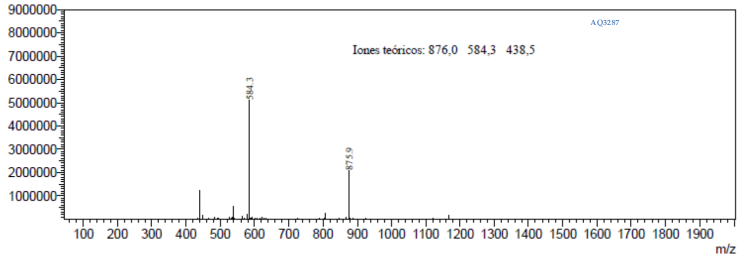
AQ3288	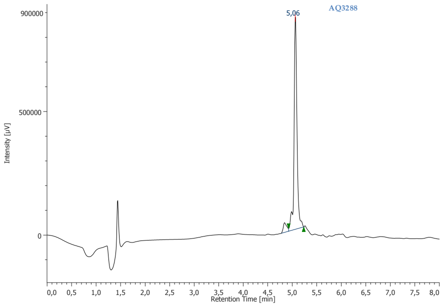	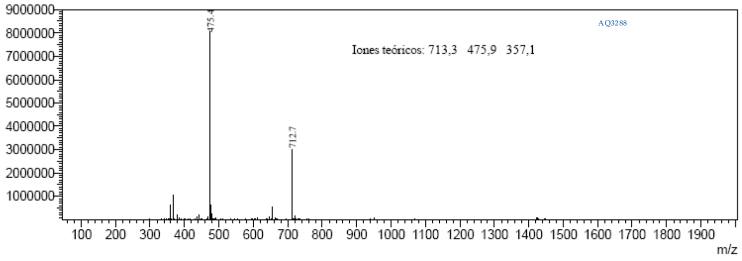
AQ3289	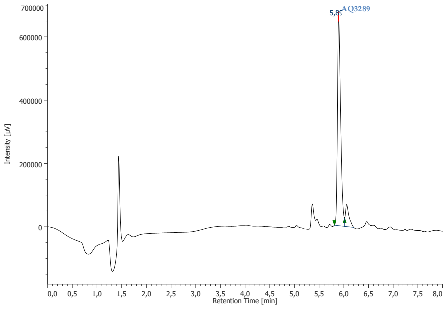	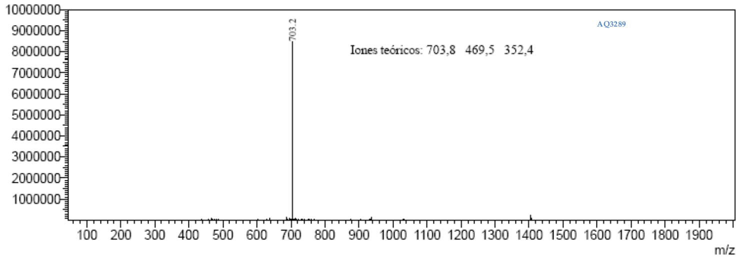

AQ3290	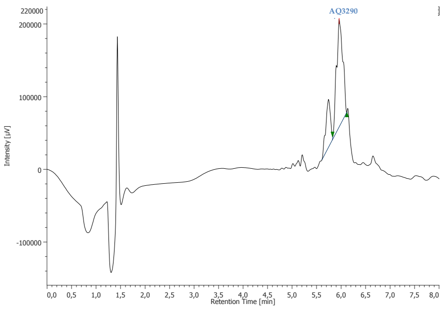	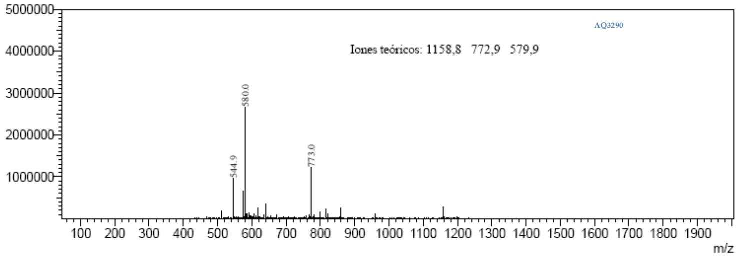
AQ3291	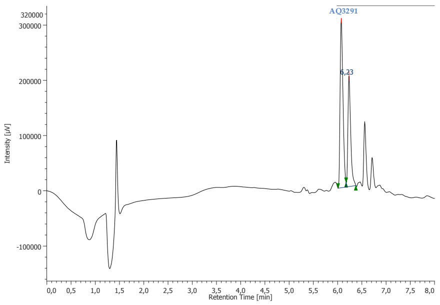	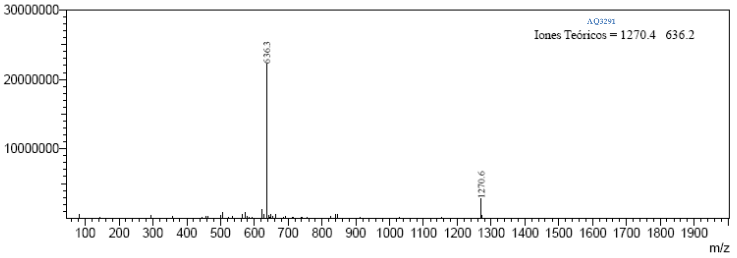
AQ3292	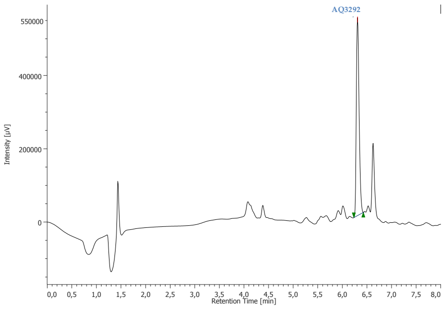	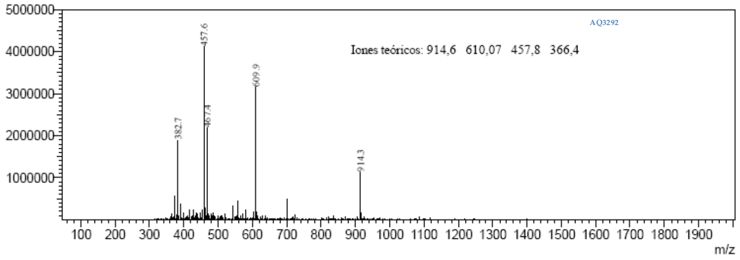
AQ3293	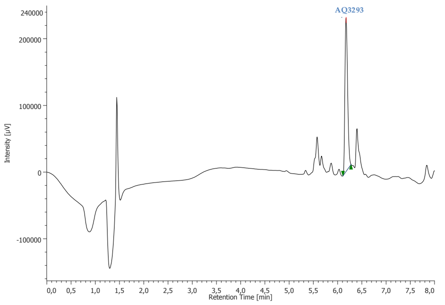	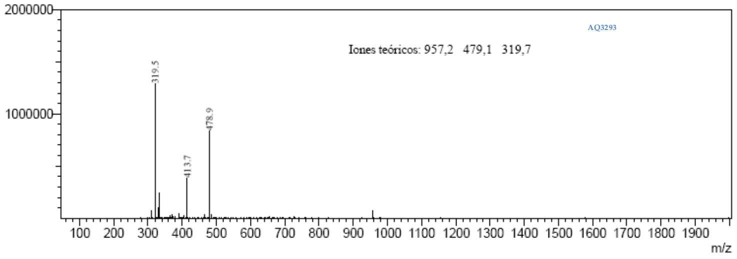

AQ3294	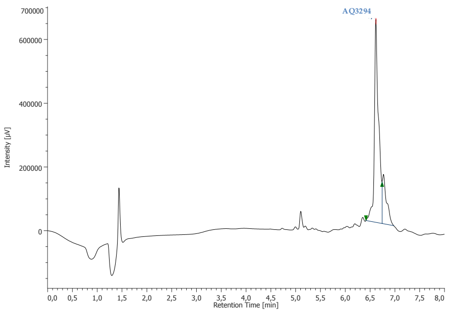	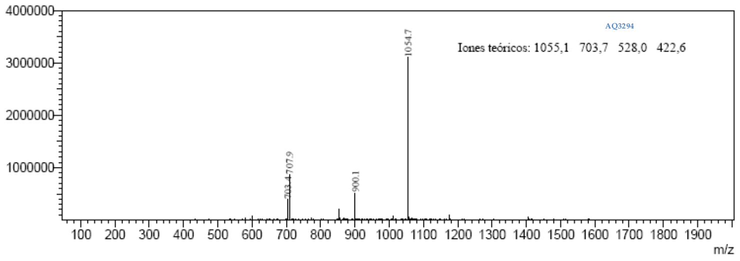
AQ3295	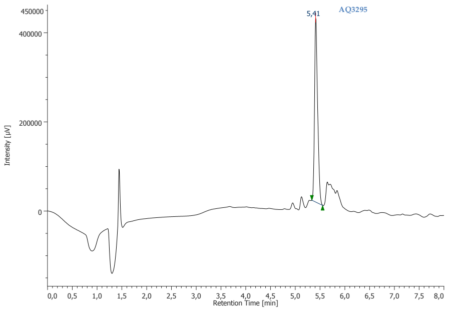	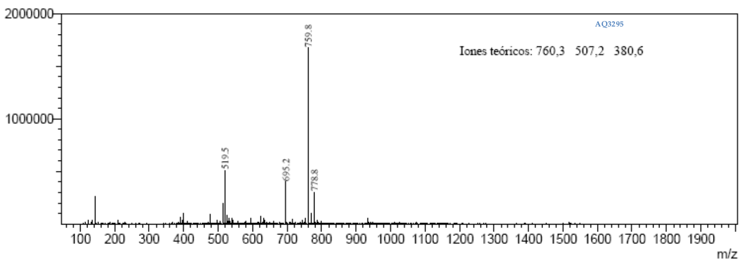
AQ3296	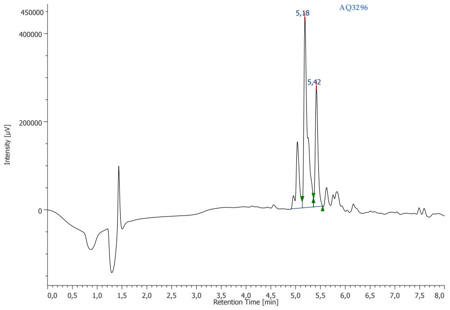	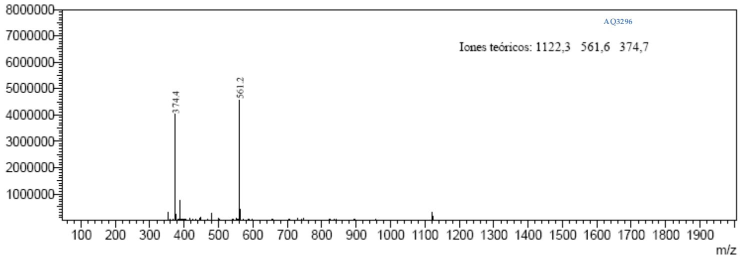
AQ3297	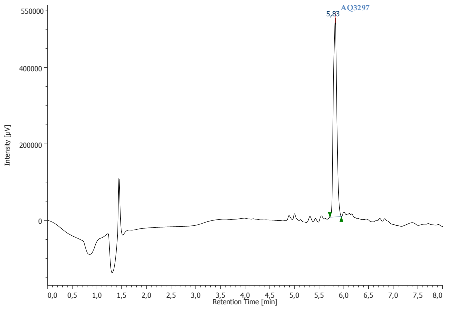	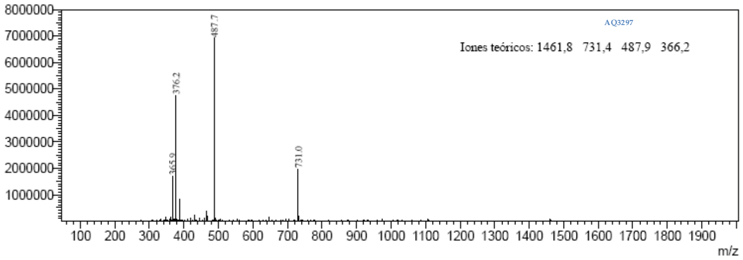

AQ3298

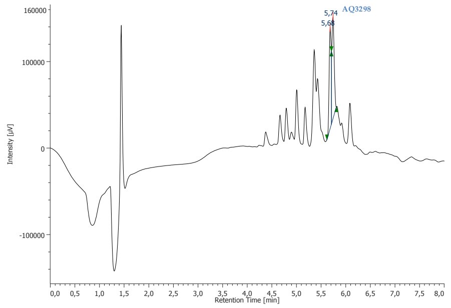

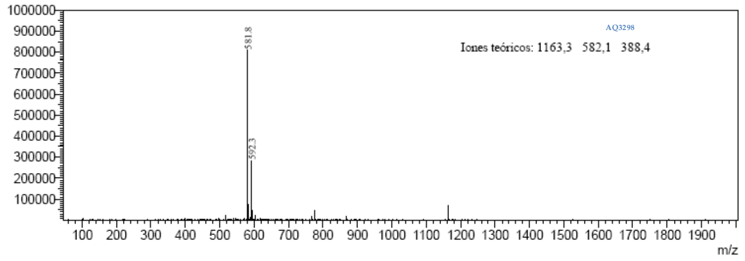

AQ3299

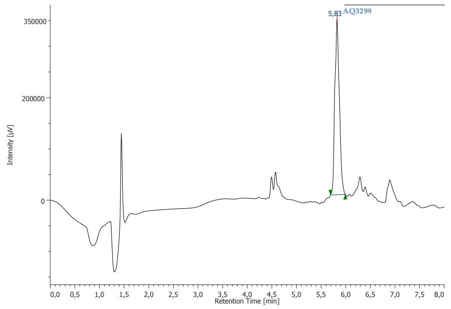

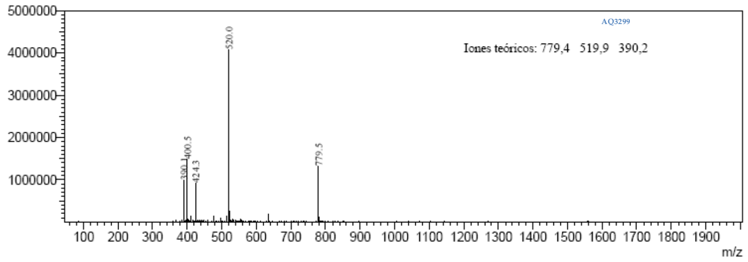

AQ3300

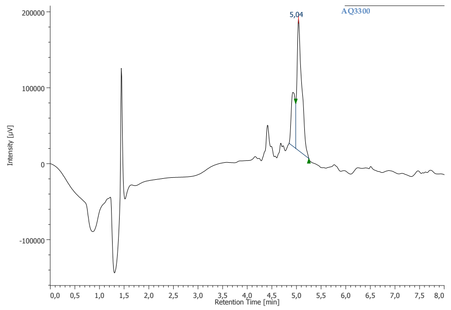

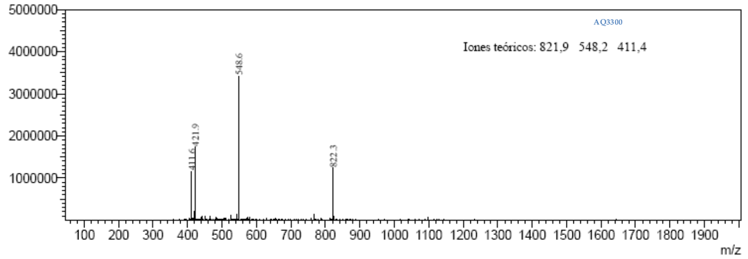

AQ3301

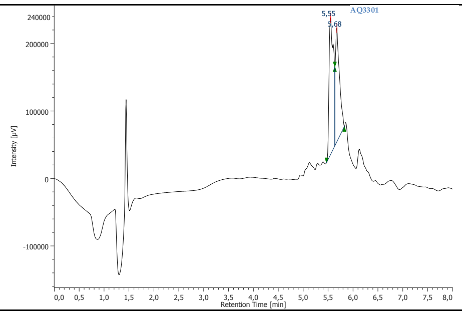

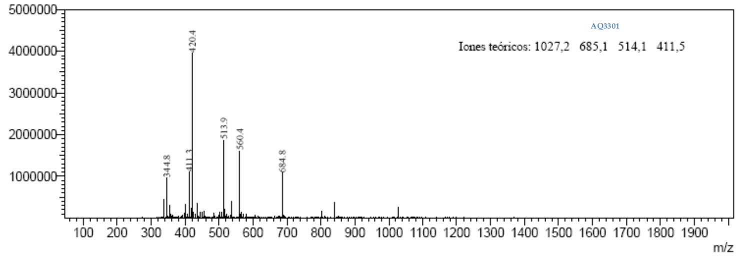

AQ3302	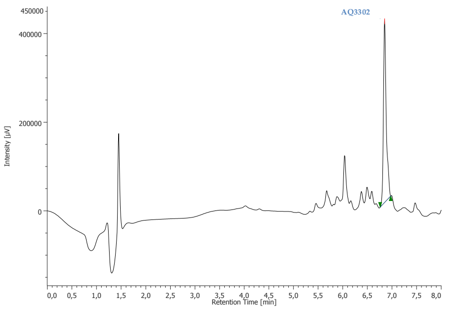	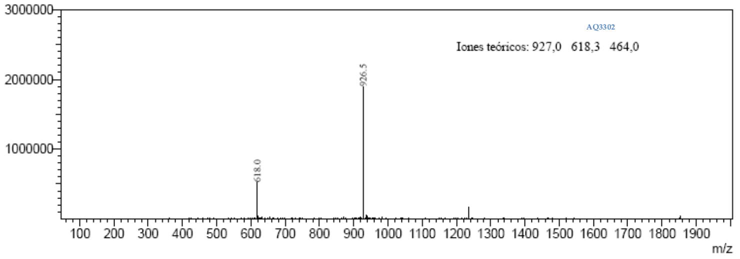
AQ3303	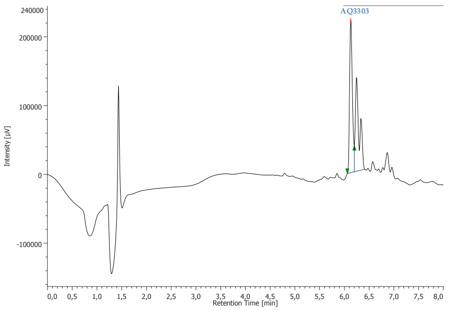	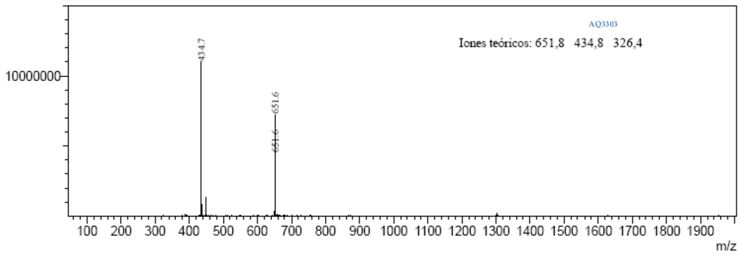
AQ3304	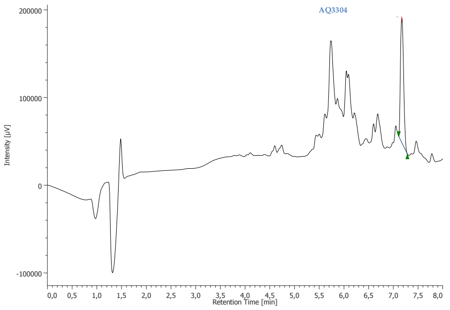	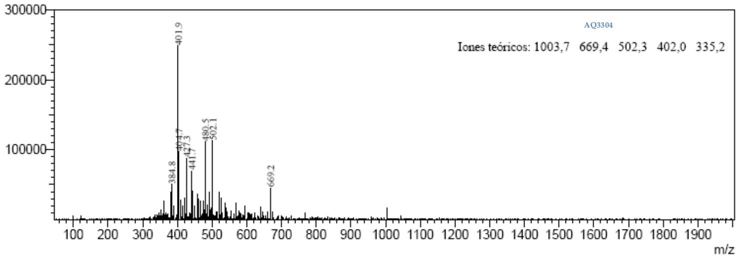
AQ3305	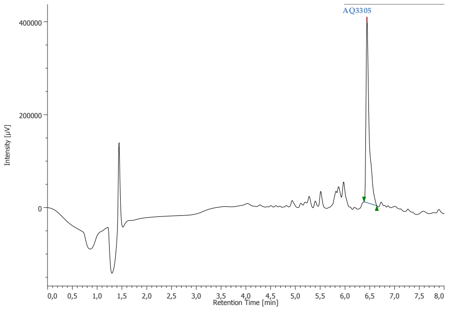	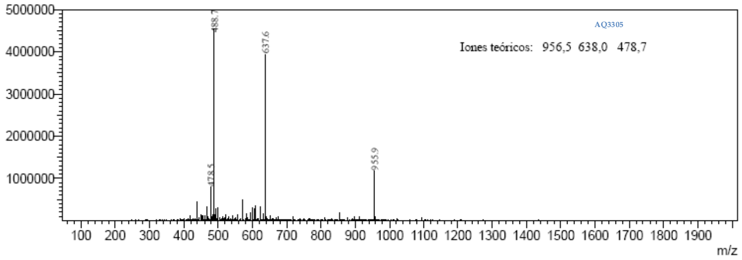

AQ3306

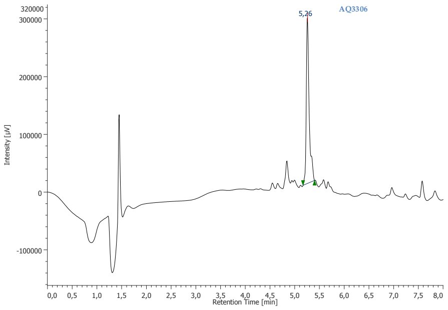

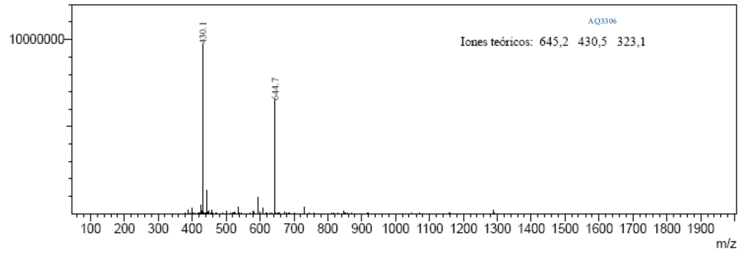

AQ3307

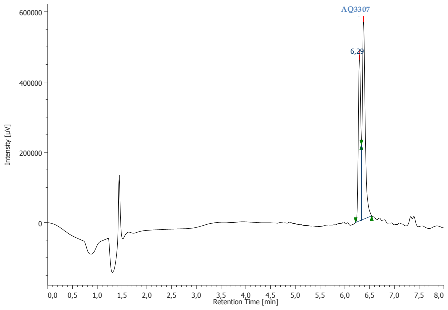

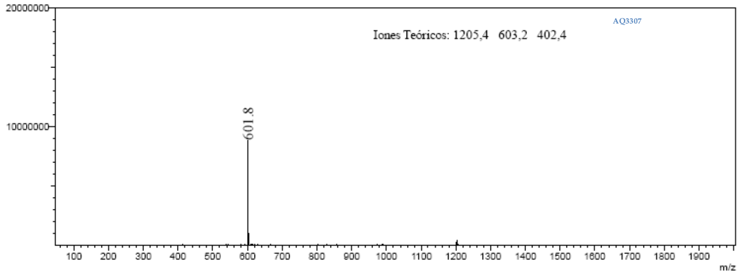

AQ3308

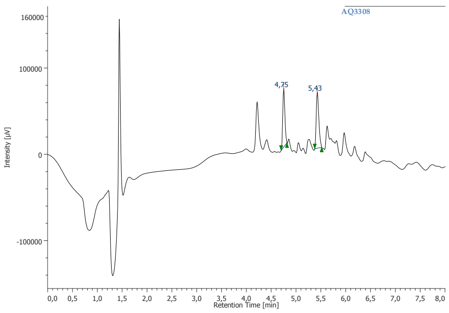

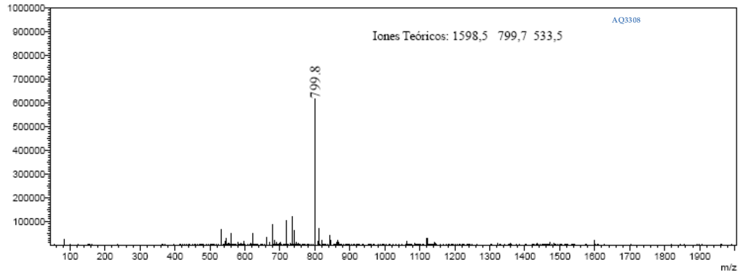

AQ3309

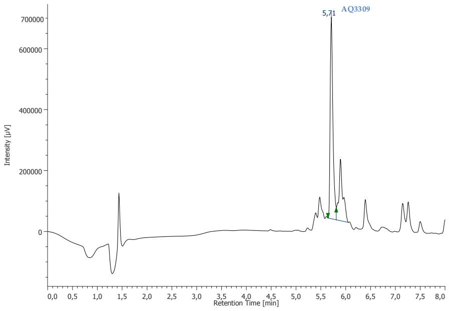

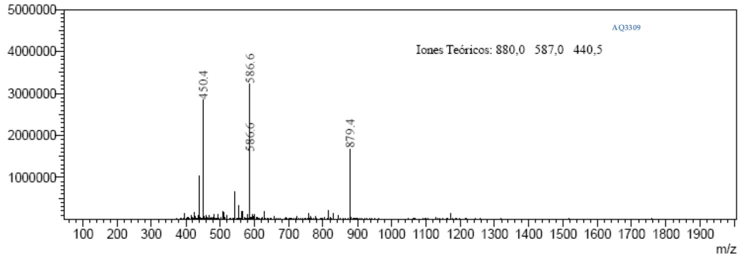

**AQ3310**	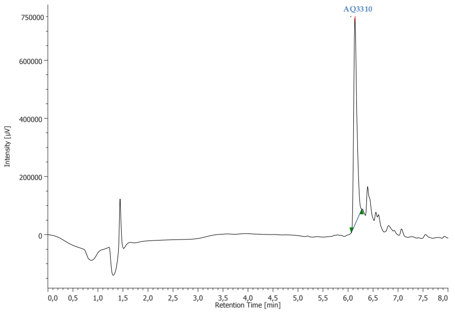	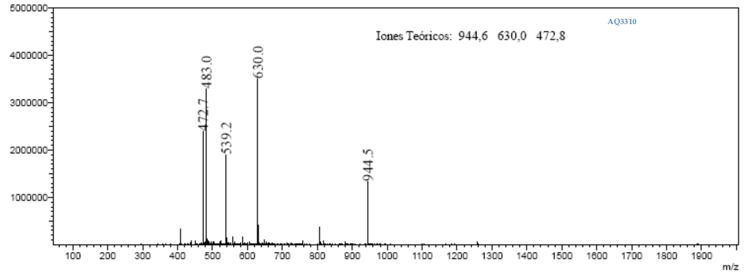
**AQ3311**	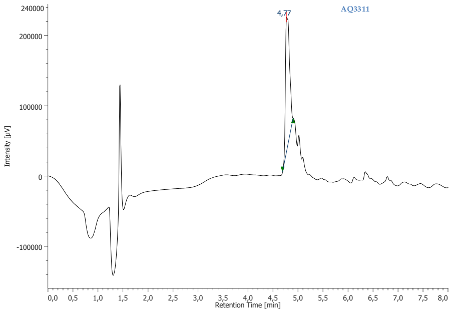	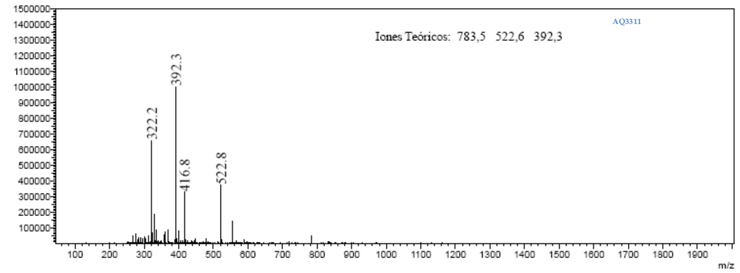
**AQ3312**	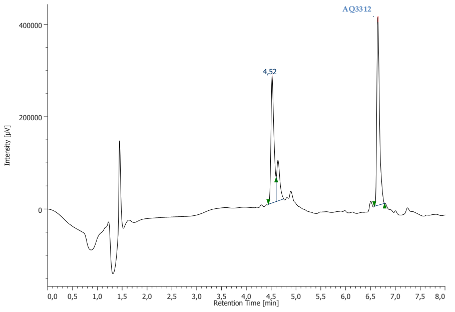	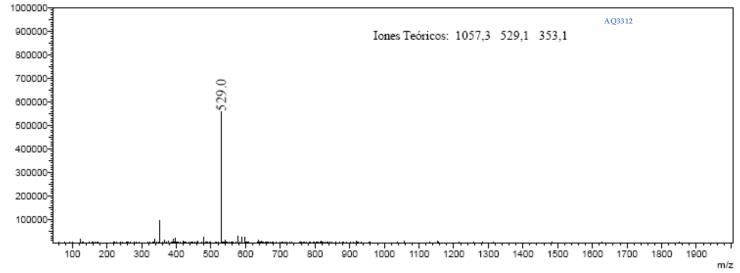
**AQ3313**	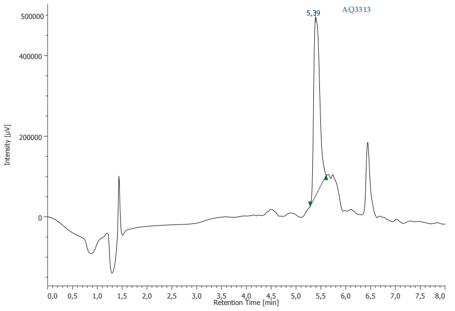	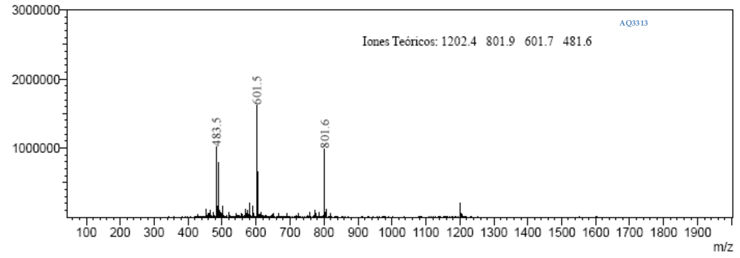

AQ3314

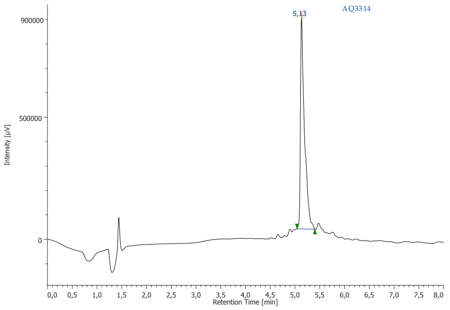

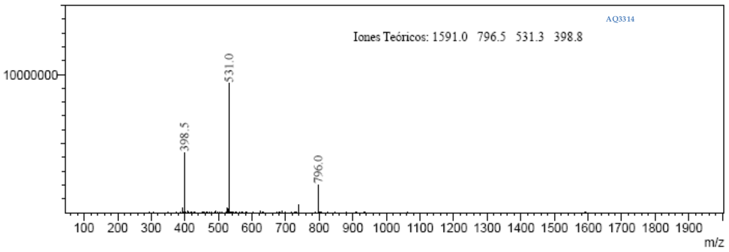

AQ3315

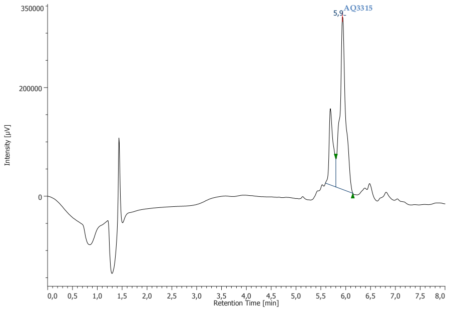

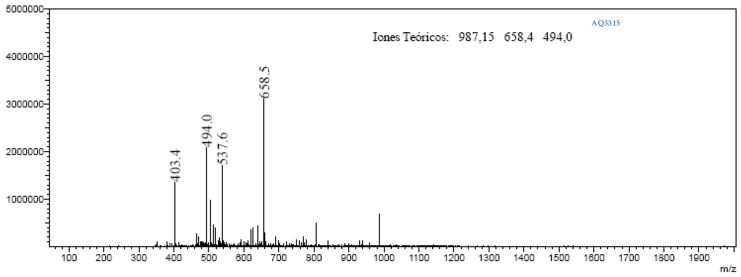

AQ3316

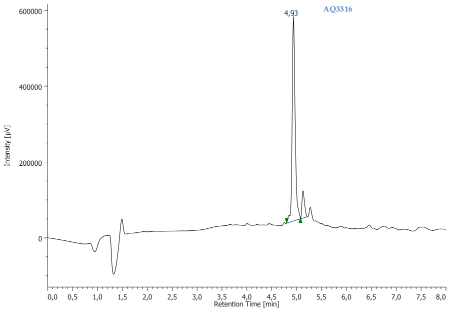

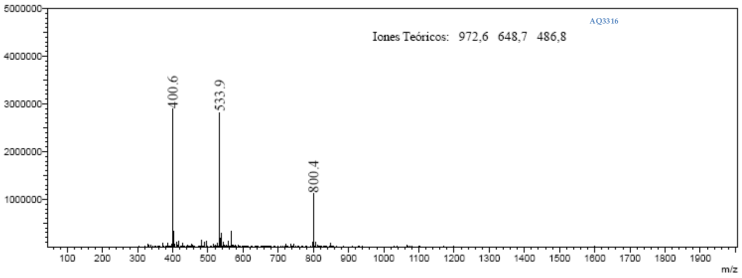

AQ3317

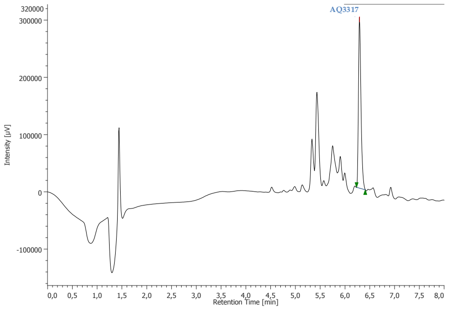

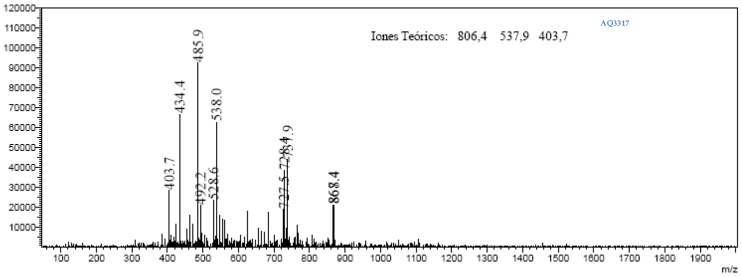

**AQ3318**	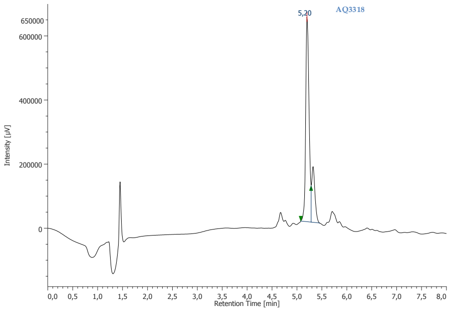	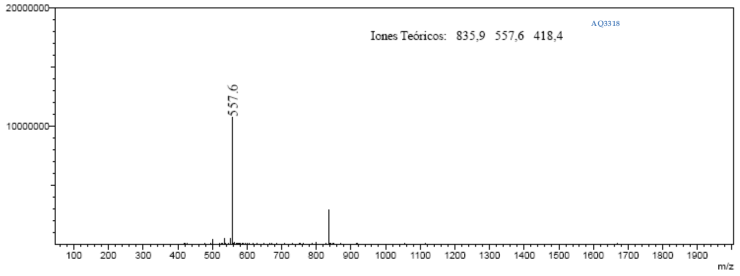
**AQ3319**	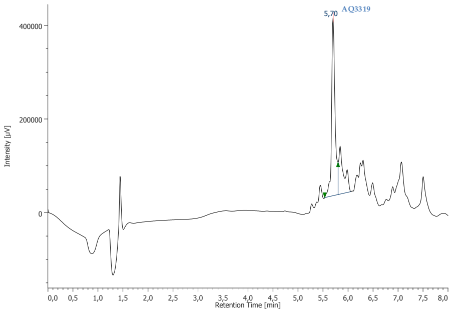	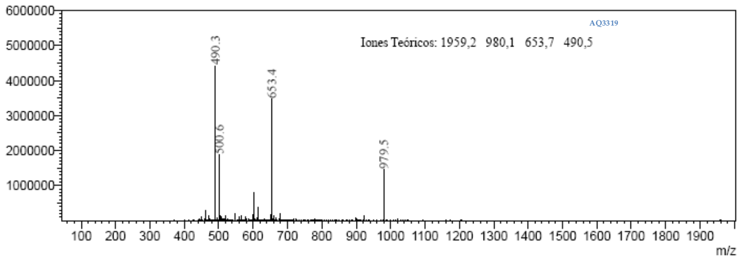
**AQ3320**	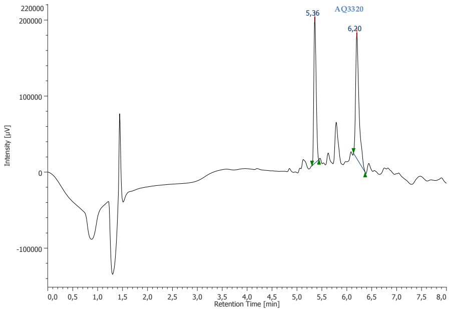	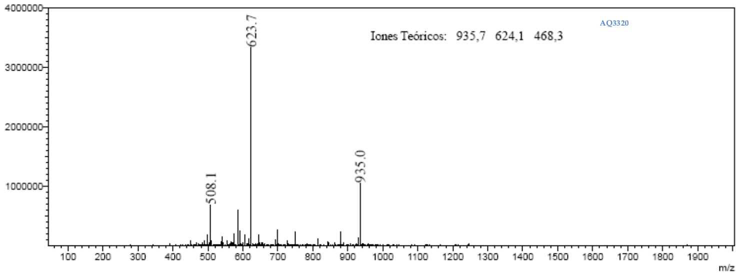
**AQ3321**	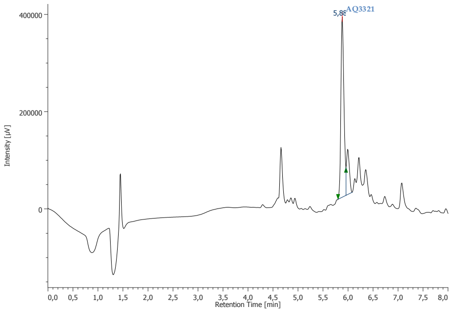	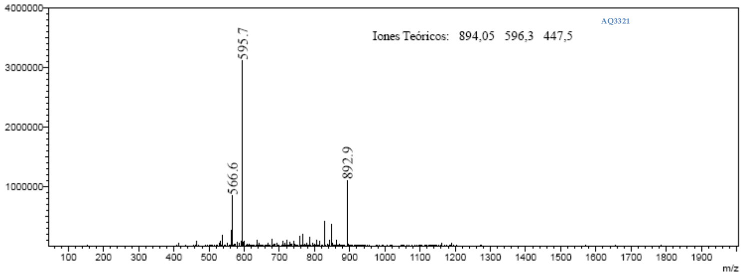

AQ3322	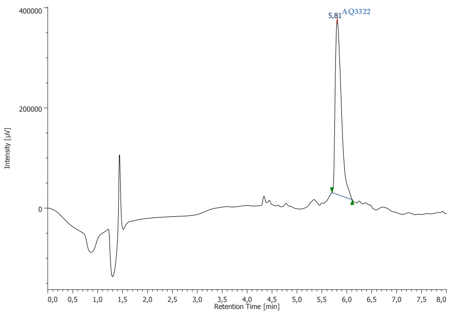	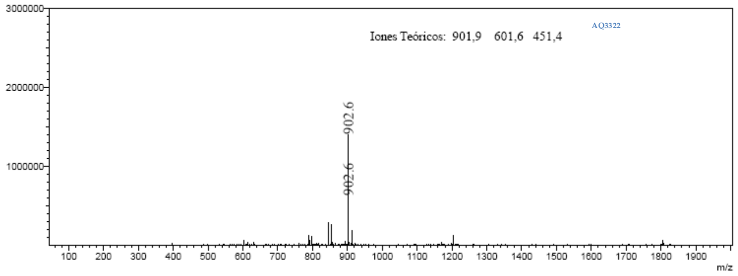
AQ3323	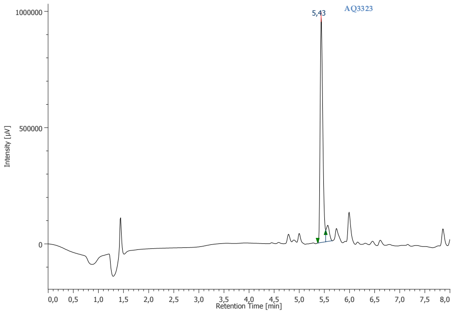	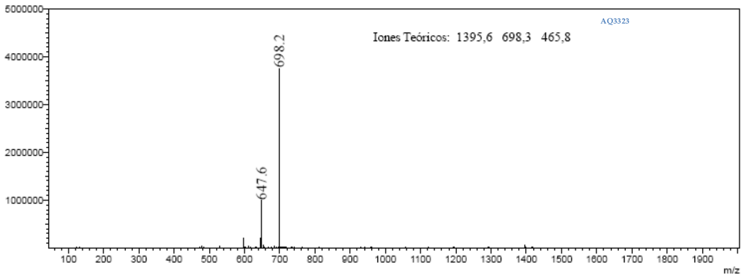
AQ3324	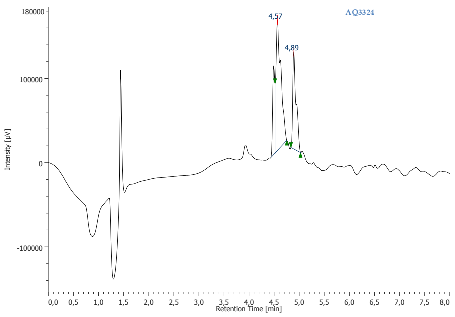	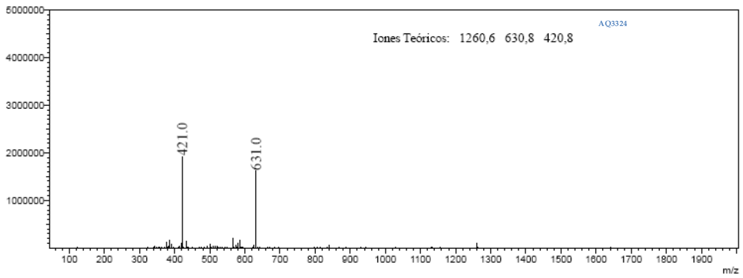
AQ3325	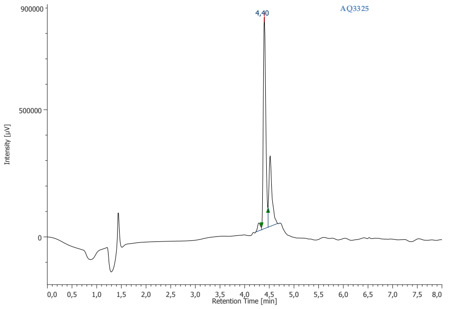	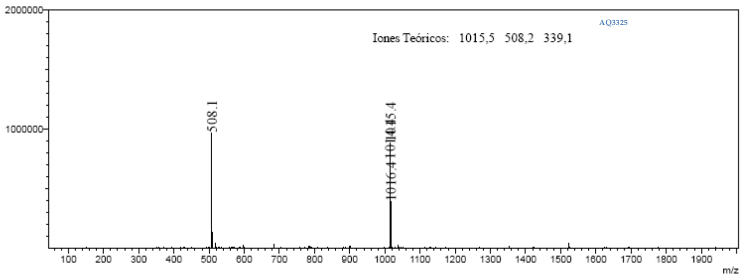

AQ3326	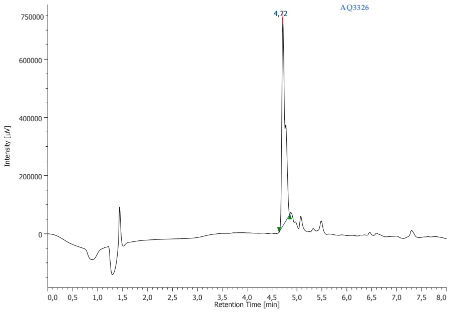	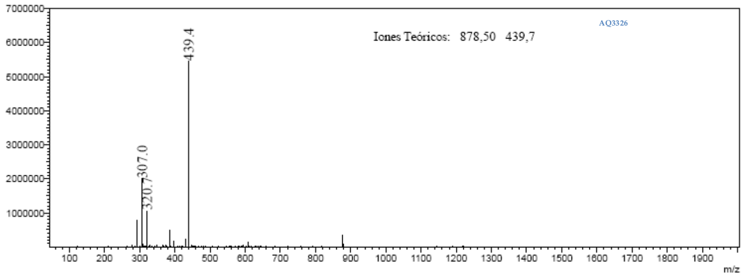
AQ3327	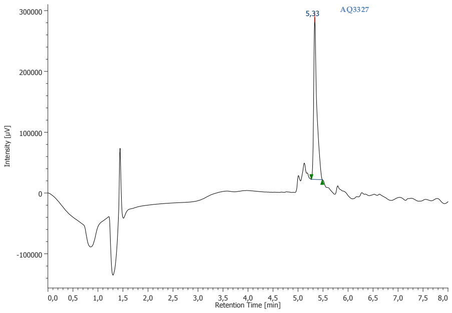	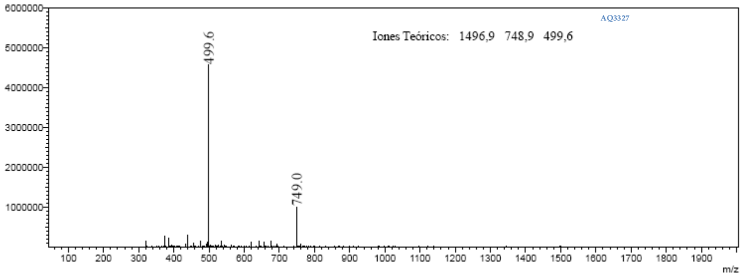
AQ3328	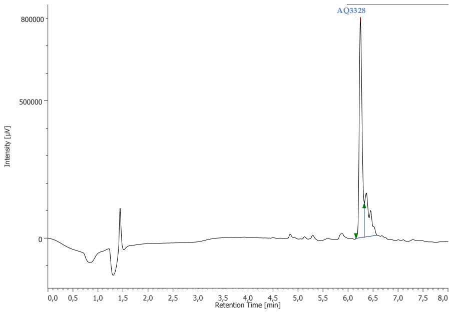	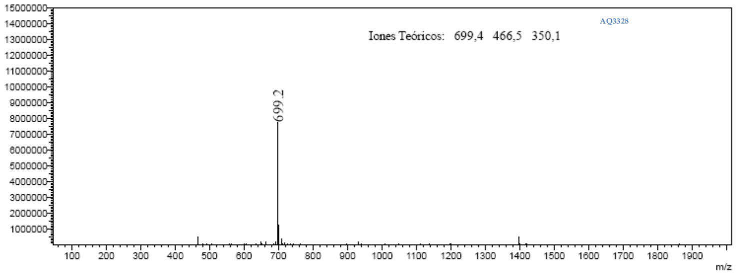
AQ3329	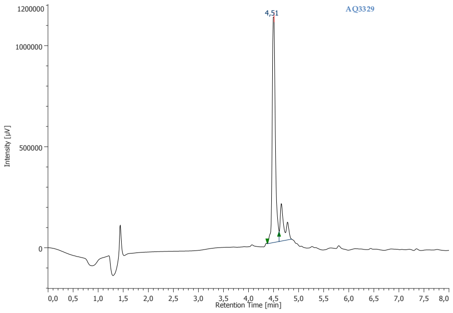	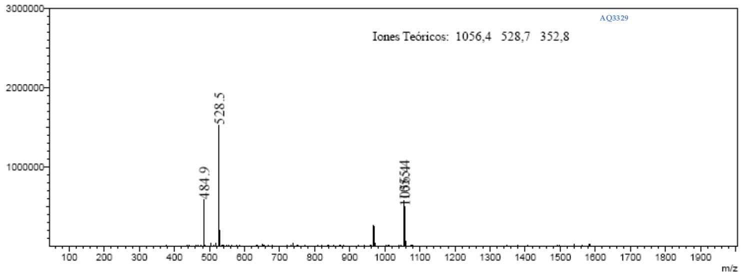

AQ3330	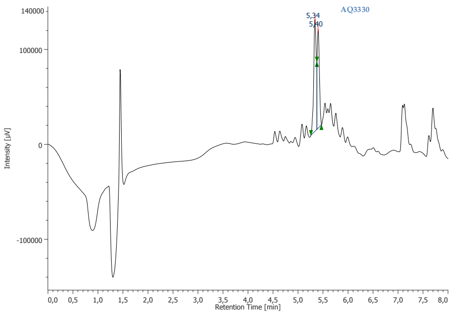	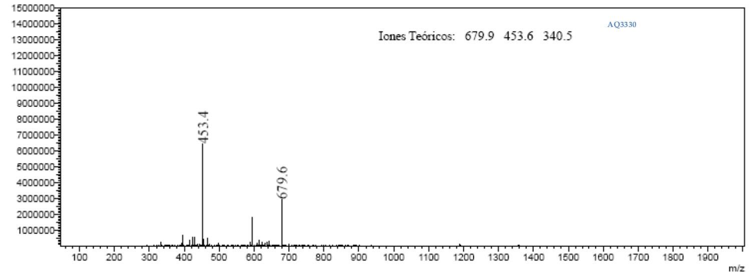
AQ3331	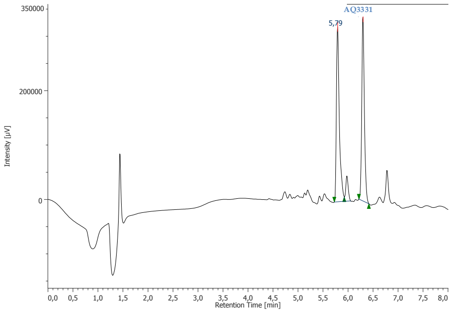	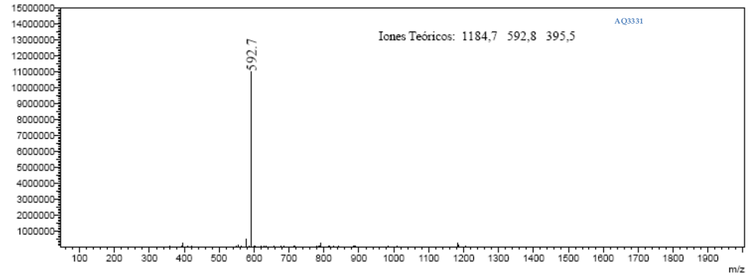
AQ3332	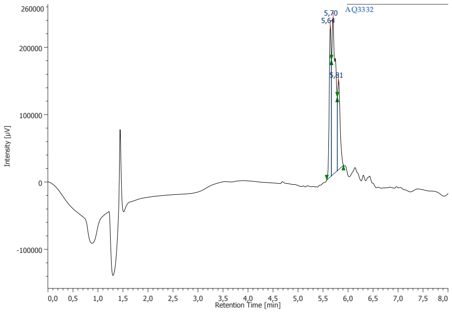	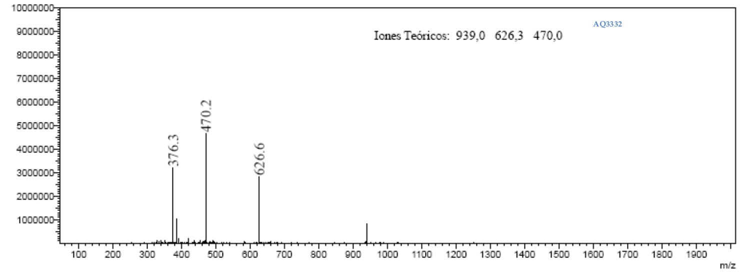
AQ3333	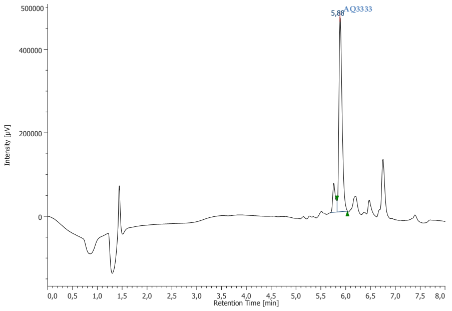	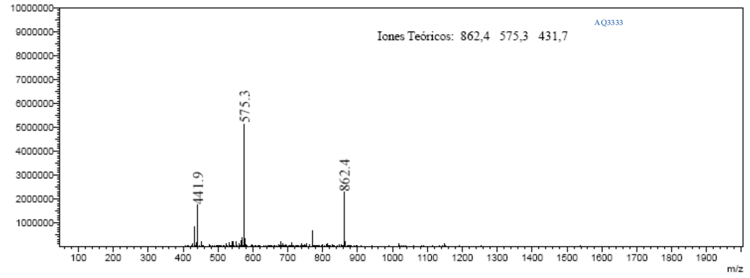

AQ3334

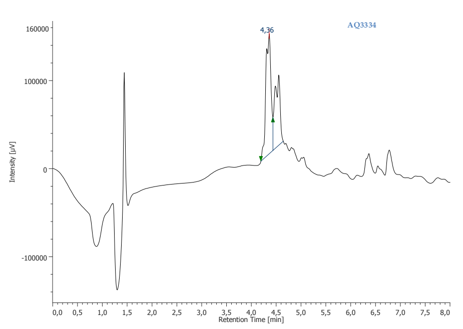

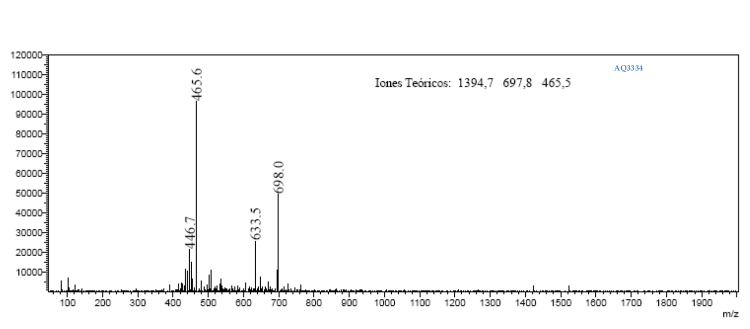

Table S2. Descriptors calculated for each peptide sequence [16].

Descriptor	Label	Definition
Aliphatic index	aIndex	Defined as the relative volume of the aliphatic side chains (A. I. L. V)
Boman Index	Boman	Potential protein interaction index. Could be associated with the potential to bind to membranes or receptors.
Charge	Charge	Theoretical net charge.
Hydrophobicity	Hphob	Gravy hydrophobicity index using Kidera scale.
Isoelectric point	pI	Isoelectric point using EMBOSS PKscale.
Length	Length	Counts the number of amino acids.
Molecular Weight	MW	Computes the molecular weight using the scales from EXpasy.

Table S3. Original variables calculated for each peptide sequence.

#	Sequence	aIndex	Boman	Charge	Hphob	pI	length	MW
AQ3283	DLKTQVKVFA	107.00	1.01	1.00	0.23	9.54	10.00	1148.37
AQ3284	VWRSTSPAKESPDELSRY	43.33	3.39	0.00	0.31	6.58	18.00	2108.30
AQ3285	ITNYLHKTLAHVPEI	130.00	0.54	0.18	-0.01	7.70	15.00	1749.04
AQ3286	TFSSKVQYQLVVTTLHKLQE	102.00	1.20	1.09	0.22	9.26	20.00	2349.71
AQ3287	RDQLPHPLKEFLQE	83.57	2.92	-0.91	0.21	5.55	14.00	1749.99
AQ3288	SMHKPLPKTLSSV	82.31	0.95	2.09	0.06	10.81	13.00	1424.72
AQ3289	EKTVIGYLEPGAE	90.00	0.86	-2.00	0.13	3.98	13.00	1405.57
AQ3290	LQRSLSPTQLYYRQFTSKV	76.84	2.41	3.00	0.22	10.65	19.00	2315.66
AQ3291	ELDSIPEKLLD	141.82	1.85	-3.00	0.11	3.74	11.00	1271.43
AQ3292	SLIRLGAFFYKRVKE	104.00	1.59	3.00	0.10	10.89	15.00	1827.20
AQ3293	QLRKVLSL	182.50	1.33	2.00	0.17	11.65	8.00	956.20
AQ3294	FYPSIQLPAHIQEYLSDS	92.22	0.96	-1.91	-0.13	4.18	18.00	2108.33
AQ3295	EAELSSDKLTISPE	90.71	2.21	-3.00	0.28	3.78	14.00	1518.64
AQ3296	DLASKRAVDF	88.00	2.58	0.00	0.21	6.45	10.00	1121.26
AQ3297	AVGKSSLVLRFVKG	125.00	0.30	3.00	0.05	11.82	14.00	1460.78
AQ3298	RGAQAAIVVYD	115.45	0.90	0.00	0.06	6.33	11.00	1162.31
AQ3299	NNRLPLLGKYIQK	120.00	1.87	3.00	0.19	10.90	13.00	1556.87
AQ3300	LNEKVSNLQRRVDA	104.29	3.82	1.00	0.52	9.69	14.00	1641.85
AQ3301	SLRDLVSRKYLHLKNIP	131.76	2.31	3.09	0.18	10.89	17.00	2052.45
AQ3302	NEDVLDQVEQSLLGVHG	120.00	1.65	-3.91	0.17	3.74	17.00	1851.99
AQ3303	VREQLQIFLR	146.00	2.60	1.00	0.23	10.40	10.00	1301.55
AQ3304	LKRKRAVLKSYLSTSVR	108.82	2.92	6.00	0.45	12.27	17.00	2005.44
AQ3305	SDSSVEVLAEELIKHRV	125.88	2.11	-1.91	0.29	4.58	17.00	1911.14
AQ3306	LKEISLKGNSLS	130.00	1.18	1.00	0.20	9.54	12.00	1288.51
AQ3307	TIPKLFQLQD	117.00	1.02	0.00	0.02	6.34	10.00	1202.42
AQ3308	QLDYNYVGDVGVEQ	90.00	1.67	-3.00	0.15	3.38	14.00	1598.69
AQ3309	AKLEVEHLKLTYSNI	130.00	1.11	0.09	0.17	7.54	15.00	1758.05
AQ3310	IEKGIQSEHFQKIALF	103.75	0.97	0.09	0.12	7.55	16.00	1888.20
AQ3311	KLKPKVKQKSIRI	112.31	2.52	6.00	0.62	12.05	13.00	1566.01
AQ3312	HTGSVRSLSN	68.00	2.91	1.09	0.32	10.55	10.00	1057.13
AQ3313	SPTDVHKQYAIVFKTPPYHS	53.50	1.46	1.18	0.07	9.13	20.00	2315.61
AQ3314	QQHKVLDKLNHLSQTP	91.25	2.47	1.18	0.34	9.54	16.00	1886.14
AQ3315	TPFDLTKSQKVRDLLDP	85.88	2.62	0.00	0.23	6.52	17.00	1973.26
AQ3316	GLNLAAHYNVFVEVVLAD	146.11	-0.37	-1.91	-0.22	4.18	18.00	1944.22
AQ3317	KYQPRLHIVEVTE	104.62	2.24	0.09	0.30	7.54	13.00	1611.86
AQ3318	RPSPPAFADDQLAASK	49.38	2.19	0.00	0.08	6.45	16.00	1670.84
AQ3319	YFKLENIYVKELSVGR	109.38	1.45	1.00	0.15	9.12	16.00	1958.29
AQ3320	GSVGLPLLKSDDIKLVNT	140.56	0.56	0.00	0.01	6.45	18.00	1869.19
AQ3321	TPVKSSKLDVFSEVYS	78.75	1.43	0.00	0.17	6.49	16.00	1786.01
AQ3322	DPGLVSANFPVSGSVQTE	75.56	0.93	-2.00	0.00	3.55	18.00	1803.94

AQ3323	TTAYFQSSYLKS	40.83	1.42	1.00	0.12	9.15	12.00	1395.53
AQ3324	SNQKVLRESEA	70.91	3.85	0.00	0.63	6.53	11.00	1260.37
AQ3325	NDTVSAPSQP	39.00	2.44	-1.00	0.20	3.75	10.00	1015.04
AQ3326	RPSRYLS	55.71	4.55	2.00	0.31	11.15	7.00	878.00
AQ3327	NLIKSKRIGQVLQ	142.31	1.73	3.00	0.34	11.82	13.00	1496.82
AQ3328	TPSSLLSPILQNQ	120.00	0.83	0.00	-0.05	6.10	13.00	1397.59
AQ3329	SPSESYSVSN	29.00	2.66	-1.00	0.24	3.85	10.00	1056.05
AQ3330	AIRSLSLQKTQL	138.33	1.62	2.00	0.24	11.65	12.00	1357.62
AQ3331	QAVAAAVASKIVG	135.38	-0.96	1.00	-0.02	9.70	13.00	1184.40
AQ3332	KSRFTVKPYIKRLQL	97.33	2.42	5.00	0.33	11.77	15.00	1877.31
AQ3333	ARDFVEKAFRDGLIS	84.67	2.58	0.00	0.19	6.56	15.00	1723.95
AQ3334	KGSESQKSSQTLD	30.00	3.69	0.00	0.66	6.49	13.00	1394.46

Table S4. Importance of components.

	PC1	PC2	PC3	PC4	PC5	PC6	PC7
Standard deviation	1.5771	1.3903	1.3226	0.75170	0.41894	0.26349	0.14319
Proportion of Variance	0.3553	0.2761	0.2499	0.08072	0.02507	0.00992	0.00293
Cumulative Proportion	0.3553	0.6314	0.8814	**0.96208**	0.9871	0.99707	1.00000

Table S5. Absolute values for the PCA factor loadings. Loadings are in scale of red to blue from higher to lower values.

	**PC1**		**PC2**		**PC3**		**PC4**
Hphob	0,51	Charge	0,52	aIndex	0,52	aIndex	0,77
Boman	0,45	pI	0,50	MW	0,45	Hphob	0,46
Charge	0,39	MW	0,46	Boman	0,44	Charge	0,29
pI	0,38	length	0,41	length	0,43	Boman	0,24
length	0,37	aIndex	0,28	Hphob	0,31	pI	0,20
MW	0,29	Boman	0,15	pI	0,22	MW	0,12
aIndex	0,13	Hphob	0,01	Charge	0,12	length	0,05

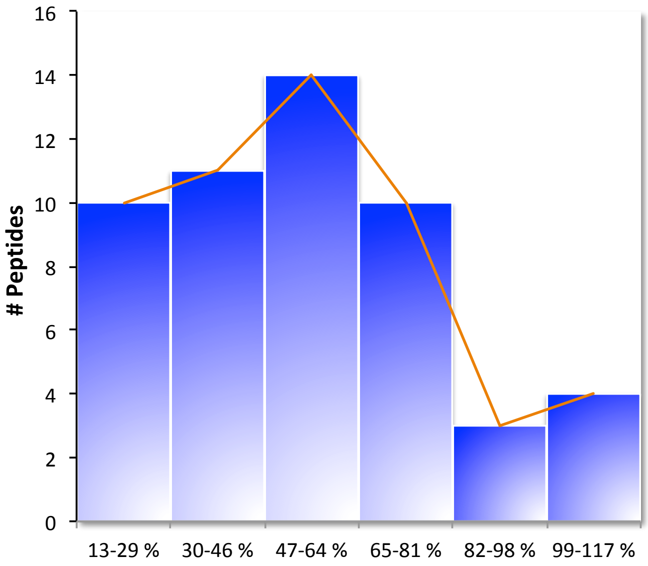

Figure S1. Number of peptides grouped by frequency according to the obtained pure yield.
