# Supplementary material for: Tea Bags for Fmoc Solid-Phase Peptide Synthesis: An Example of Circular Economy
Source: Molecules. 2021 Aug 19;26(16):5035. doi: 10.3390/molecules26165035 (PMC8399505; doi:10.3390/molecules26165035)
Supplement: Supplementary file 1 [file molecules-26-05035-s001.zip › Work_Document.pdf]

Synthesis Name: S14062019

Amount of Synthesis: 40 mg.

Deprotection Method: 4MP 20% TritonX100 1%/DMF

Number of Peptides: 52

Number of families: 22

-

Bag	Size	Pos.	M.W.	Family
1	10	442	1148.5	RAG-1
Sequence: DLKTQVKVFA				
2	18	927	2108.52	RAG-1
Sequence: VWRSTSPAKESPDELSRY				
3	15	967	1749.26	RAG-1
Sequence: ITNYLHKTLAHVPEI				
4	20	28	2350	SOCS3
Sequence: TFSSKVQYQLVVTTLHKLQE				
5	14	194	1750.17	SOCS3
Sequence: RDQLPHPLKEFLQE				
6	13	1	1424.89	IFNRa/b2
Sequence: SMHKPLPKTLSSV				
7	13	177	1405.75	IFNRa/b2
Sequence: EKTVIGYLEPGAE				
8	19	142	2315.91	IFNRa/b2
Sequence: LQRSLSPTQLYYRQFTSKV				
9	11	31	1271.56	IFIT5
Sequence: ELDSIPEKLLD				
10	15	146	1827.4	IFIT5
Sequence: SLIRLGAFFYKRVKE				
11	8	205	956.29	IFIT5
Sequence: QLRKVLSL				
12	18	267	2108.58	IFNR1B
Sequence: FYPSIQLPAHIQEYLSDS				
13	14	299	1518.81	IFNR1B
Sequence: EAELSSDKLTISPE				
14	10	1	1121.37	RAB5
Sequence: DLASKRAVDF				
15	14	1	1460.97	RAB5
Sequence: AVGKSSLVLRFVKG				
16	11	1	1162.46	RAB5
Sequence: RGAQAAIVVYD				

17 13 1 1557.05 TLR4
Sequence: NNRLPLLGKYIQK

18 14 1 1642.03 MCR2
Sequence: LNEKVSNLQRRVDA

19 17 227 2052.67 NLRX-1
Sequence: SLRDLVSRKYLHLKNIP

20 17 612 1852.23 NLRX-1
Sequence: NEDVLDQVEQSLLGVHG

21 10 956 1301.69 NLRX-1
Sequence: VREQLQIFLR

22 17 613 2005.65 NOD-1
Sequence: LKRKRAVLKSYLSTSVR

23 17 742 1911.37 NOD-1
Sequence: SDSSVEVLAEELIKHRV

24 12 927 1288.66 NOD-1
Sequence: LKEISLKGNSLS

25 10 613 1202.55 NOD-2
Sequence: TIPKLFQLQD

26 14 781 1598.89 NOD-2
Sequence: QLDYNYVGDVGVEQ

27 15 745 1758.25 NOD-2
Sequence: AKLEVEHLKLTYSNI

28 16 821 1888.44 NOD-2
Sequence: IEKGIQSEHFQKIALF

29 13 235 1566.2 GPR-18
Sequence: KLKPKVKQKSIRI

30 10 333 1057.26 GPR-18
Sequence: HTGSVRSLSN

31 20 284 2315.9 NF-Kb
Sequence: SPTDVHKQYAIVFKTPPYHS

32 16 512 1886.37 F-kb
Sequence: QQHKVLDKLNHLSQTP

33 17 747 1973.47 F-kb
Sequence: TPFDLTKSQKVRDLLDP

34 18 247 1944.46 EOMES
Sequence: GLNLAAHYNVFVEVVLAD

35 13 336 1612.05 EOMES
Sequence: KYQPRLHIVEVTE

36 17 1 1742.12 EOMES
Sequence: RPSPPAFADDQLAASAK

37 16 69 1958.51 1
Sequence: YFKLENIYVKELSVGR

38 18 405 1869.43 1
Sequence: GSVGLPLLKSDDIKLVNT

39 16 102 1786.22 protein-like
Sequence: TPVKSSKLDVFSEVYS

40 18 173 1804.19 IL12R
Sequence: DPGLVSANFPVSGSVQTE

41 12 743 1395.68 IL12R
Sequence: TTAYFQSSYLKS

42 11 71 1260.51 TNFR
Sequence: SNQKVLRESEA

43 10 1 1015.17 TNFR
Sequence: NDTVSAPSQP

44 7 1 878.07 TNFR
Sequence: RPSRYLS

45 13 65 1497.01 IL15R
Sequence: NLIKSKRIGQVLQ

46 13 124 1397.76 IL15R
Sequence: TPSSLLSPILQNQ

47 10 256 1056.17 IL15R
Sequence: SPSESYSVSN

48 12 258 1357.77 TLR3
Sequence: AIRSLSLQKTQL

49 13 29 1184.58 PSMA7
Sequence: QAVAAAVASKIVG

50 15 54 1877.51 PSMA7
Sequence: KSRFTVKPYIKRLQL

51 15 99 1724.14 1
Sequence: ARDFVEKAFRDGLIS

52 13 1 1394.63 1
Sequence: KGSESQKSSQTLD

1	DLKTQVKVFA
2	VWRSTSPAKESPDELSRY
3	ITNYLHKTLAHVPEI
4	TFSSKVQYQLVVTTLHKLQE
5	RDQLPHPLKEFLQE
6	SMHKPLPKTLSSV
7	EKTVIGYLEPGAE
8	LQRSLSPTQLYYRQFTSKV
9	ELDSIPEKLLD
10	SLIRLGAFFYKRVKE
11	QLRKVLSL
12	FYPSIQLPAHIQEYLSDS
13	EAELSSDKLTISPE
14	DLASKRAVDF
15	AVGKSSLVLRFVKG
16	RGAQAAIVVYD
17	NNRLPLLGKYIQK
18	LNEKVSNLQRRVDA
19	SLRDLVSRKYLHLKNIP
20	NEDVLDQVEQSLLGVHG
21	VREQLQIFLR
22	LKRKRAVLKSYLSTSVR
23	SDSSVEVLAEELIKHRV
24	LKEISLKGNSLS
25	TIPKLFQLQD
26	QLDYNYVGDVGVEQ
27	AKLEVEHLKLTYSNI
28	IEKGIQSEHFQKIALF
29	KLKPKVKQKSIRI
30	HTGSVRSLSN
31	SPTDVHKQYAIVFKTPPYHS
32	QQHKVLDKLNHLSQTP

33	TPFDLTKSQKVRDLLDP
34	GLNLAAHYNVFVEVVLAD
35	KYQPRLHIVEVTE
36	RPSPPAFADDQLAASAK
37	YFKLENIYVKELSVGR
38	GSVGLPLLKSDDIKLVNT
39	TPVKSSKLDVFSEVYS
40	DPGLVSANFPVSGSVQTE
41	TTAYFQSSYLKS
42	SNQKVLRESEA
43	NDTVSAPSQP
44	RPSRYLS
45	NLIKSKRIGQVLQ
46	TPSSLLSPILQNQ
47	SPSESYSVSN
48	AIRSLSLQKTQL
49	QAVAAAVASKIVG
50	KSRFTVKPYIKRLQL
51	ARDFVEKAFRDGLIS
52	KGSESQKSSQTLD

TOTAL OF AMINO ACIDS FOR SYNTHESIS

ALA	39	RESIDUOS
ARG	37	RESIDUOS
ASN	22	RESIDUOS
ASP	34	RESIDUOS
CYS	0	RESIDUOS
PHE	24	RESIDUOS
GLY	24	RESIDUOS
GLU	41	RESIDUOS
GLN	47	RESIDUOS
HIS	18	RESIDUOS
ILE	32	RESIDUOS
LEU	96	RESIDUOS
LYS	66	RESIDUOS
MET	1	RESIDUOS
PRO	38	RESIDUOS
SER	86	RESIDUOS
TYR	28	RESIDUOS
THR	32	RESIDUOS
TRP	1	RESIDUOS
VAL	65	RESIDUOS

AMOUNT OF AMINO ACIDS FROM COUPLING 1 TO 10

ALA	31	RESIDUES
ARG	27	RESIDUES
ASN	18	RESIDUES
ASP	22	RESIDUES
CYS	0	RESIDUES
PHE	17	RESIDUES
GLY	15	RESIDUES
GLU	26	RESIDUES
GLN	32	RESIDUES
HIS	11	RESIDUES
ILE	18	RESIDUES
LEU	70	RESIDUES
LYS	52	RESIDUES
MET	1	RESIDUES
PRO	30	RESIDUES
SER	63	RESIDUES
TYR	20	RESIDUES
THR	21	RESIDUES
TRP	1	RESIDUES
VAL	40	RESIDUES
OUT	9	RESIDUES

AMOUNT OF AMINO ACIDS FROM COUPLING 11 TO 20

ALA	8	RESIDUES
ARG	10	RESIDUES
ASN	4	RESIDUES
ASP	12	RESIDUES
CYS	0	RESIDUES
PHE	7	RESIDUES
GLY	9	RESIDUES
GLU	15	RESIDUES
GLN	15	RESIDUES
HIS	7	RESIDUES
ILE	14	RESIDUES
LEU	26	RESIDUES
LYS	14	RESIDUES
MET	0	RESIDUES
PRO	8	RESIDUES
SER	23	RESIDUES
TYR	8	RESIDUES
THR	11	RESIDUES
TRP	0	RESIDUES
VAL	25	RESIDUES
OUT	43	RESIDUES

COUPLING NUMBER 1

ALA:	1 18 42 =**3**
ASP:	9 16 25 34 52 =**5**
GLU:	4 5 7 10 13 35 40 =**7**
PHE:	14 28 =**2**
GLY:	15 20 49 =**3**
ILE:	3 27 29 =**3**
LYS:	17 36 =**2**
LEU:	11 48 50 =**3**
ASN:	30 47 =**2**
PRO:	19 32 33 43 =**4**
GLN:	26 45 46 =**3**
ARG:	21 22 37 =**3**
SER:	12 24 31 39 41 44 51 =**7**
THR:	38 =**1**
VAL:	6 8 23 =**3**
TYR:	2 =**1**

DEPROTECTION DATE____/___/____	CHECK	MADE BY	REVIEWED BY
4MP 20% TritonX100 1%/DMF(2x10')	|____|___|	________________	________________
Wash DMF(3x1')	|__|__|__|	________________	________________
Wash IPA(1x1')	|_________|	________________	________________
Wash BPB 1%/DMF(1x2')	|_________|	________________	________________
Wash DMF(2x1')	|____|___|	________________	________________
Wash DCM(1x1')	|_________|	________________	________________

Coupling Cycle	DATE	HOUR	MADE BY	REVIEWED BY
AA+HBTU+OXYMA+DIPEA Single (5:5:5:7,5)	|___/___/___|	__:__|__:__|	________________	________________
AA+HCTU+OXYMA+DIPEA Double (5:5:5:7,5)	|___/___/___|	__:__|__:__|	________________	________________
AA+DIC+OXYMA Triple (5:5:5)	|___/___/___|	__:__|__:__|	________________	________________

After coupling cycle, before checking the BPB test, wash DMF(2x1')

1:ALA	16:ASP	31:SER	46:GLN
2:TYR	17:LYS	32:PRO	47:ASN
3:ILE	18:ALA	33:PRO	48:LEU
4:GLU	19:PRO	34:ASP	49:GLY
5:GLU	20:GLY	35:GLU	50:LEU
6:VAL	21:ARG	36:LYS	51:SER
7:GLU	22:ARG	37:ARG	52:ASP
8:VAL	23:VAL	38:THR	
9:ASP	24:SER	39:SER	
10:GLU	25:ASP	40:GLU	
11:LEU	26:GLN	41:SER	
12:SER	27:ILE	42:ALA	
13:GLU	28:PHE	43:PRO	
14:PHE	29:ILE	44:SER	
15:GLY	30:ASN	45:GLN	

COUPLING NUMBER 2

ALA:	7 34 36 =**3**
ASP:	12 14 18 33 =**4**
GLU:	3 26 42 =**3**
PHE:	1 =**1**
GLY:	37 =**1**
HIS:	20 31 =**2**
ILE:	19 51 =**2**
LYS:	8 10 15 41 =**4**
LEU:	9 21 24 28 44 45 52 =**7**
ASN:	27 38 46 =**3**
PRO:	13 =**1**
GLN:	4 5 17 25 43 48 50 =**7**
ARG:	2 23 29 =**3**
SER:	6 11 30 47 =**4**
THR:	32 35 40 =**3**
VAL:	22 49 =**2**
TYR:	16 39 =**2**

DEPROTECTION	DATE____/___/____	CHECK	MADE BY	REVIEWED BY
4MP 20% TritonX100 1%/DMF(2x10')		|_____|___|	________________	________________
Wash DMF(3x1')		|__|__|__|	________________	________________
Wash IPA(1x1')		|_________|	________________	________________
Wash BPB 1%/DMF(1x2')		|_________|	________________	________________
Wash DMF(2x1')		|_____|___|	________________	________________
Wash DCM(1x1')		|_________|	________________	________________

Coupling Cycle	DATE	HOUR	MADE BY	REVIEWED BY
AA+HBTU+OXYMA+DIPEA Single (5:5:5:7,5)	|___/___/___|	|__:__|__:__|	________________	________________
AA+HCTU+OXYMA+DIPEA Double (5:5:5:7,5)	|___/___/___|	|__:__|__:__|	________________	________________
AA+DIC+OXYMA Triple (5:5:5)	|___/___/___|	|__:__|__:__|	________________	________________

After coupling cycle, before checking the BPB test, wash DMF(2x1')

1:PHE	16:TYR	31:HIS	46:ASN
2:ARG	17:GLN	32:THR	47:SER
3:GLU	18:ASP	33:ASP	48:GLN
4:GLN	19:ILE	34:ALA	49:VAL
5:GLN	20:HIS	35:THR	50:GLN
6:SER	21:LEU	36:ALA	51:ILE
7:ALA	22:VAL	37:GLY	52:LEU
8:LYS	23:ARG	38:ASN	
9:LEU	24:LEU	39:TYR	
10:LYS	25:GLN	40:THR	
11:SER	26:GLU	41:LYS	
12:ASP	27:ASN	42:GLU	
13:PRO	28:LEU	43:GLN	
14:ASP	29:ARG	44:LEU	
15:LYS	30:SER	45:LEU	

COUPLING NUMBER 3

```
ALA:    28 =1
PHE:    21 =1
GLY:    7 =1
HIS:    23 =1
ILE:    17 29 49 =3
LEU:    4 5 9 11 25 30 33 34 41 50 51 =11
ASN:    19 =1
PRO:    3 =1
GLN:    32 40 46 =3
SER:    2 6 8 12 13 22 24 27 36 42 43 =11
THR:    48 52 =2
VAL:    1 10 14 15 16 18 20 26 35 37 38 39 45 47 =14
TYR:    31 44 =2
```

DEPROTECTION DATE____/___/____ CHECK MADE BY REVIEWED BY

4MP 20% TritonX100 1%/DMF(2x10') |_____|___|________________ ________________

Wash DMF(3x1') |__|__|__|________________ ________________

Wash IPA(1x1') |_________|________________ ________________

Wash BPB 1%/DMF(1x2') |_________|________________ ________________

Wash DMF(2x1') |_____|___|________________ ________________

Wash DCM(1x1') |_________|________________ ________________

Coupling Cycle DATE HOUR MADE BY REVIEWED BY

AA+HBTU+OXYMA+DIPEA
Single (5:5:5:7,5) |___/___/___|__:__|__:__|________________ ________________

AA+HCTU+OXYMA+DIPEA
Double (5:5:5:7,5) |___/___/___|__:__|__:__|________________ ________________

AA+DIC+OXYMA
Triple (5:5:5) |___/___/___|__:__|__:__|________________ ________________

After coupling cycle, before checking the BPB test, wash DMF(2x1')

```
1:VAL   16:VAL  31:TYR  46:GLN
2:SER   17:ILE  32:GLN  47:VAL
3:PRO   18:VAL  33:LEU  48:THR
4:LEU   19:ASN  34:LEU  49:ILE
5:LEU   20:VAL  35:VAL  50:LEU
6:SER   21:PHE  36:SER  51:LEU
7:GLY   22:SER  37:VAL  52:THR
8:SER   23:HIS  38:VAL
9:LEU   24:SER  39:VAL
10:VAL  25:LEU  40:GLN
11:LEU  26:VAL  41:LEU
12:SER  27:SER  42:SER
13:SER  28:ALA  43:SER
14:VAL  29:ILE  44:TYR
15:VAL  30:LEU  45:VAL
```

COUPLING NUMBER 4

ALA:	14 36 =	**2**
GLU:	35 39 42 =	**3**
PHE:	5 15 =	**2**
GLY:	20 26 51 =	**3**
ILE:	13 21 28 =	**3**
LYS:	1 4 9 19 23 48 49 =	**7**
LEU:	2 6 12 33 38 46 =	**6**
ASN:	24 =	**1**
PRO:	7 31 43 =	**3**
GLN:	25 45 52 =	**3**
ARG:	10 18 44 50 =	**4**
SER:	29 30 32 37 47 =	**5**
THR:	8 22 =	**2**
VAL:	3 11 16 34 40 =	**5**
TYR:	17 27 41 =	**3**

DEPROTECTION DATE____/___/____	CHECK	MADE BY	REVIEWED BY
4MP 20% TritonX100 1%/DMF(2x10')	|____|___|	__________	__________
Wash DMF(3x1')	|__|__|__|	__________	__________
Wash IPA(1x1')	|________|	__________	__________
Wash BPB 1%/DMF(1x2')	|________|	__________	__________
Wash DMF(2x1')	|____|___|	__________	__________
Wash DCM(1x1')	|________|	__________	__________

Coupling Cycle	DATE	HOUR	MADE BY	REVIEWED BY
AA+HBTU+OXYMA+DIPEA<br>Single (5:5:5:7,5)	|___/___/___|	__:__|__:__|	__________	__________
AA+HCTU+OXYMA+DIPEA<br>Double (5:5:5:7,5)	|___/___/___|	__:__|__:__|	__________	__________
AA+DIC+OXYMA<br>Triple (5:5:5)	|___/___/___|	__:__|__:__|	__________	__________

After coupling cycle, before checking the BPB test, wash DMF(2x1')

1:LYS	16:VAL	31:PRO	46:LEU
2:LEU	17:TYR	32:SER	47:SER
3:VAL	18:ARG	33:LEU	48:LYS
4:LYS	19:LYS	34:VAL	49:LYS
5:PHE	20:GLY	35:GLU	50:ARG
6:LEU	21:ILE	36:ALA	51:GLY
7:PRO	22:THR	37:SER	52:GLN
8:THR	23:LYS	38:LEU	
9:LYS	24:ASN	39:GLU	
10:ARG	25:GLN	40:VAL	
11:VAL	26:GLY	41:TYR	
12:LEU	27:TYR	42:GLU	
13:ILE	28:ILE	43:PRO	
14:ALA	29:SER	44:ARG	
15:PHE	30:SER	45:GLN	

COUPLING NUMBER 5

ALA: 36 43 =**2**
ASP: 33 51 =**2**
GLU: 2 5 7 9 =**4**
PHE: 8 25 =**2**
GLY: 24 45 =**2**
HIS: 3 4 =**2**
ILE: 16 23 46 =**3**
LYS: 10 11 17 28 29 38 50 =**7**
LEU: 19 20 32 37 =**4**
PRO: 31 =**1**
GLN: 21 48 =**2**
ARG: 14 15 18 30 42 =**5**
SER: 22 39 40 41 44 49 52 =**7**
THR: 6 13 27 =**3**
VAL: 1 26 34 35 =**4**
TYR: 12 47 =**2**

DEPROTECTION	DATE____/___/____	CHECK	MADE BY	REVIEWED BY
4MP 20% TritonX100 1%/DMF(2x10')		|_____|___|	________________	________________
Wash DMF(3x1')		|__|__|__|	________________	________________
Wash IPA(1x1')		|_________|	________________	________________
Wash BPB 1%/DMF(1x2')		|_________|	________________	________________
Wash DMF(2x1')		|_____|___|	________________	________________
Wash DCM(1x1')		|_________|	________________	________________

Coupling Cycle	DATE	HOUR	MADE BY	REVIEWED BY
AA+HBTU+OXYMA+DIPEA Single (5:5:5:7,5)	|___/___/___|	__:__|__:__|	________________	________________
AA+HCTU+OXYMA+DIPEA Double (5:5:5:7,5)	|___/___/___|	__:__|__:__|	________________	________________
AA+DIC+OXYMA Triple (5:5:5)	|___/___/___|	__:__|__:__|	________________	________________

After coupling cycle, before checking the BPB test, wash DMF(2x1')

1:VAL	16:ILE	31:PRO	46:ILE
2:GLU	17:LYS	32:LEU	47:TYR
3:HIS	18:ARG	33:ASP	48:GLN
4:HIS	19:LEU	34:VAL	49:SER
5:GLU	20:LEU	35:VAL	50:LYS
6:THR	21:GLN	36:ALA	51:ASP
7:GLU	22:SER	37:LEU	52:SER
8:PHE	23:ILE	38:LYS	
9:GLU	24:GLY	39:SER	
10:LYS	25:PHE	40:SER	
11:LYS	26:VAL	41:SER	
12:TYR	27:THR	42:ARG	
13:THR	28:LYS	43:ALA	
14:ARG	29:LYS	44:SER	
15:ARG	30:ARG	45:GLY	

COUPLING NUMBER 6

ALA:	3 16 49 =**3**
ASP:	2 26 =**2**
GLU:	12 34 37 =**3**
PHE:	39 =**1**
GLY:	17 40 =**2**
HIS:	19 32 =**2**
ILE:	35 38 45 50 =**4**
LYS:	5 6 14 24 =**4**
LEU:	4 7 13 15 20 21 22 23 25 27 36 42 48 =**13**
PRO:	9 44 46 =**3**
GLN:	1 8 18 28 29 =**5**
ARG:	11 33 51 =**3**
SER:	41 43 47 52 =**4**
THR:	31 =**1**
VAL:	30 =**1**
TYR:	10 =**1**

DEPROTECTION DATE____/___/____ CHECK MADE BY REVIEWED BY

4MP 20% TritonX100 1%/DMF(2x10') |_____|___|_________________ _________________

Wash DMF(3x1') |__|__|__|_________________ _________________

Wash IPA(1x1') |_________|_________________ _________________

Wash BPB 1%/DMF(1x2') |_________|_________________ _________________

Wash DMF(2x1') |_____|___|_________________ _________________

Wash DCM(1x1') |_________|_________________ _________________

Coupling Cycle DATE HOUR MADE BY REVIEWED BY

AA+HBTU+OXYMA+DIPEA
Single (5:5:5:7,5) |___/___/___|__:__|__:__|_________________ _________________

AA+HCTU+OXYMA+DIPEA
Double (5:5:5:7,5) |___/___/___|__:__|__:__|_________________ _________________

AA+DIC+OXYMA
Triple (5:5:5) |___/___/___|__:__|__:__|_________________ _________________

After coupling cycle, before checking the BPB test, wash DMF(2x1')

1:GLN	16:ALA	31:THR	46:PRO
2:ASP	17:GLY	32:HIS	47:SER
3:ALA	18:GLN	33:ARG	48:LEU
4:LEU	19:HIS	34:GLU	49:ALA
5:LYS	20:LEU	35:ILE	50:ILE
6:LYS	21:LEU	36:LEU	51:ARG
7:LEU	22:LEU	37:GLU	52:SER
8:GLN	23:LEU	38:ILE	
9:PRO	24:LYS	39:PHE	
10:TYR	25:LEU	40:GLY	
11:ARG	26:ASP	41:SER	
12:GLU	27:LEU	42:LEU	
13:LEU	28:GLN	43:SER	
14:LYS	29:GLN	44:PRO	
15:LEU	30:VAL	45:ILE	

COUPLING NUMBER 7

ALA:	16 =**1**
ASP:	38 =**1**
GLU:	23 47 =**2**
PHE:	10 28 51 =**3**
GLY:	26 =**1**
HIS:	35 =**1**
ILE:	9 =**1**
LYS:	13 25 27 29 31 37 52 =**7**
LEU:	3 5 11 17 18 19 24 =**7**
ASN:	32 =**1**
PRO:	2 6 =**2**
GLN:	12 21 36 41 =**4**
ARG:	8 44 45 =**3**
SER:	14 20 30 40 46 48 =**6**
THR:	1 4 =**2**
VAL:	15 33 34 39 42 43 49 =**7**
TYR:	7 22 50 =**3**

DEPROTECTION DATE____/___/____ CHECK MADE BY REVIEWED BY

4MP 20% TritonX100 1%/DMF(2x10') |_____|___|________________ ________________

Wash DMF(3x1') |__|__|__|________________ ________________

Wash IPA(1x1') |_________|________________ ________________

Wash BPB 1%/DMF(1x2') |_________|________________ ________________

Wash DMF(2x1') |_____|___|________________ ________________

Wash DCM(1x1') |_________|________________ ________________

Coupling Cycle DATE HOUR MADE BY REVIEWED BY

AA+HBTU+OXYMA+DIPEA
Single (5:5:5:7,5) |___/___/___|__:__|__:__|________________ ________________

AA+HCTU+OXYMA+DIPEA
Double (5:5:5:7,5) |___/___/___|__:__|__:__|________________ ________________

AA+DIC+OXYMA
Triple (5:5:5) |___/___/___|__:__|__:__|________________ ________________

After coupling cycle, before checking the BPB test, wash DMF(2x1')

1:THR	16:ALA	31:LYS	46:SER
2:PRO	17:LEU	32:ASN	47:GLU
3:LEU	18:LEU	33:VAL	48:SER
4:THR	19:LEU	34:VAL	49:VAL
5:LEU	20:SER	35:HIS	50:TYR
6:PRO	21:GLN	36:GLN	51:PHE
7:TYR	22:TYR	37:LYS	52:LYS
8:ARG	23:GLU	38:ASP	
9:ILE	24:LEU	39:VAL	
10:PHE	25:LYS	40:SER	
11:LEU	26:GLY	41:GLN	
12:GLN	27:LYS	42:VAL	
13:LYS	28:PHE	43:VAL	
14:SER	29:LYS	44:ARG	
15:VAL	30:SER	45:ARG	

COUPLING NUMBER 8

ALA: 14 49 51 =**3**
ASP: 13 36 38 39 =**4**
GLU: 21 23 =**2**
PHE: 10 31 34 41 =**4**
GLY: 7 30 =**2**
HIS: 28 =**1**
ILE: 12 =**1**
LYS: 1 33 42 45 =**4**
LEU: 6 15 17 27 32 35 46 48 =**8**
ASN: 18 =**1**
PRO: 5 25 50 =**3**
GLN: 11 16 20 52 =**4**
SER: 2 9 22 24 47 =**5**
THR: 3 4 43 =**3**
VAL: 26 29 37 40 =**4**
TYR: 8 19 =**2**
OUT: 44 =**1**

DEPROTECTION DATE____/___/____ CHECK MADE BY REVIEWED BY

4MP 20% TritonX100 1%/DMF(2x10') |_____|___|________________ ________________

Wash DMF(3x1') |__|__|__|________________ ________________

Wash IPA(1x1') |_________|________________ ________________

Wash BPB 1%/DMF(1x2') |_________|________________ ________________

Wash DMF(2x1') |_____|___|________________ ________________

Wash DCM(1x1') |_________|________________ ________________

Coupling Cycle DATE HOUR MADE BY REVIEWED BY

AA+HBTU+OXYMA+DIPEA
Single (5:5:5:7,5) |___/___/___|__:__|__:__|________________ ________________

AA+HCTU+OXYMA+DIPEA
Double (5:5:5:7,5) |___/___/___|__:__|__:__|________________ ________________

AA+DIC+OXYMA
Triple (5:5:5) |___/___/___|__:__|__:__|________________ ________________

After coupling cycle, before checking the BPB test, wash DMF(2x1')

1:LYS 16:GLN 31:PHE 46:LEU
2:SER 17:LEU 32:LEU 47:SER
3:THR 18:ASN 33:LYS 48:LEU
4:THR 19:TYR 34:PHE 49:ALA
5:PRO 20:GLN 35:LEU 50:PRO
6:LEU 21:GLU 36:ASP 51:ALA
7:GLY 22:SER 37:VAL 52:GLN
8:TYR 23:GLU 38:ASP
9:SER 24:SER 39:ASP
10:PHE 25:PRO 40:VAL
<u>11:GLN</u> 26:VAL 41:PHE
12:ILE 27:LEU 42:LYS
13:ASP 28:HIS 43:THR
14:ALA 29:VAL 44:OUT
15:LEU 30:GLY 45:LYS

COUPLING NUMBER 9

ALA:	10 16 23 49 =**4**
ASP:	9 36 43 =**3**
GLU:	2 20 28 =**3**
HIS:	5 12 27 =**3**
ILE:	7 24 25 =**3**
LYS:	3 19 22 29 32 50 51 =**7**
LEU:	1 14 39 46 =**4**
PRO:	6 17 40 47 =**4**
GLN:	33 42 =**2**
ARG:	21 35 =**2**
SER:	13 15 18 38 45 48 52 =**7**
THR:	30 =**1**
VAL:	4 31 34 =**3**
TYR:	8 26 37 41 =**4**
OUT:	11 =**1**

DEPROTECTION DATE____/___/____	CHECK	MADE BY	REVIEWED BY
4MP 20% TritonX100 1%/DMF(2x10')	|____|___|	____________	____________
Wash DMF(3x1')	|__|__|__|	____________	____________
Wash IPA(1x1')	|________|	____________	____________
Wash BPB 1%/DMF(1x2')	|________|	____________	____________
Wash DMF(2x1')	|____|___|	____________	____________
Wash DCM(1x1')	|________|	____________	____________

Coupling Cycle	DATE	HOUR	MADE BY	REVIEWED BY
AA+HBTU+OXYMA+DIPEA Single (5:5:5:7,5)	|___/___/___|	__:__|__:__|	____________	____________
AA+HCTU+OXYMA+DIPEA Double (5:5:5:7,5)	|___/___/___|	__:__|__:__|	____________	____________
AA+DIC+OXYMA Triple (5:5:5)	|___/___/___|	__:__|__:__|	____________	____________

After coupling cycle, before checking the BPB test, wash DMF(2x1')

1:LEU	16:ALA	31:VAL	47:PRO
2:GLU	17:PRO	32:LYS	48:SER
3:LYS	18:SER	33:GLN	49:ALA
4:VAL	19:LYS	34:VAL	50:LYS
5:HIS	20:GLU	35:ARG	51:LYS
6:PRO	21:ARG	36:ASP	52:SER
7:ILE	22:LYS	37:TYR	
8:TYR	23:ALA	38:SER	
9:ASP	24:ILE	39:LEU	
10:ALA	25:ILE	40:PRO	
11:OUT	26:TYR	41:TYR	
12:HIS	27:HIS	42:GLN	
13:SER	28:GLU	43:ASP	
14:LEU	29:LYS	45:SER	
15:SER	30:THR	46:LEU	

COUPLING NUMBER 10

ALA: 12 36 41 49 =**4**
ASP: 1 14 32 =**3**
GLU: 24 27 51 52 =**4**
PHE: 40 =**1**
GLY: 10 16 =**2**
HIS: 3 30 =**2**
ILE: 31 37 =**2**
LYS: 2 6 38 39 45 =**5**
LEU: 8 9 17 22 23 =**5**
ASN: 26 34 42 43 =**4**
PRO: 5 29 35 =**3**
ARG: 19 48 =**2**
SER: 13 15 28 33 46 47 =**6**
THR: 25 =**1**
VAL: 4 7 18 20 21 50 =**6**

DEPROTECTION DATE____/___/____ CHECK MADE BY REVIEWED BY

4MP 20% TritonX100 1%/DMF(2x10') |____|___|________________ ________________

Wash DMF(3x1') |__|__|__|________________ ________________

Wash IPA(1x1') |_________|________________ ________________

Wash BPB 1%/DMF(1x2') |_________|________________ ________________

Wash DMF(2x1') |____|___|________________ ________________

Wash DCM(1x1') |_________|________________ ________________

Coupling Cycle DATE HOUR MADE BY REVIEWED BY

AA+HBTU+OXYMA+DIPEA
Single (5:5:5:7,5) |___/___/___|__:__|__:__|________________ ________________

AA+HCTU+OXYMA+DIPEA
Double (5:5:5:7,5) |___/___/___|__:__|__:__|________________ ________________

AA+DIC+OXYMA
Triple (5:5:5) |___/___/___|__:__|__:__|________________ ________________

After coupling cycle, before checking the BPB test, wash DMF(2x1')

1:ASP	17:LEU	32:ASP	48:ARG
2:LYS	18:VAL	33:SER	49:ALA
3:HIS	19:ARG	34:ASN	50:VAL
4:VAL	20:VAL	35:PRO	51:GLU
5:PRO	21:VAL	36:ALA	52:GLU
6:LYS	22:LEU	37:ILE	
7:VAL	23:LEU	38:LYS	
8:LEU	24:GLU	39:LYS	
9:LEU	25:THR	40:PHE	
10:GLY	26:ASN	41:ALA	
12:ALA	27:GLU	42:ASN	
13:SER	28:SER	43:ASN	
14:ASP	29:PRO	45:LYS	
15:SER	30:HIS	46:SER	
16:GLY	31:ILE	47:SER	

COUPLING NUMBER 11

ALA:	2 31 =**2**
GLU:	9 =**1**
PHE:	36 =**1**
HIS:	6 =**1**
ILE:	45 48 =**2**
LYS:	15 18 24 29 33 =**5**
LEU:	3 4 5 10 13 32 38 =**7**
ASN:	37 40 =**2**
PRO:	12 =**1**
GLN:	8 20 28 35 =**4**
ARG:	16 17 =**2**
SER:	19 39 42 46 52 =**5**
THR:	7 41 50 =**3**
VAL:	22 23 27 49 51 =**5**
TYR:	26 34 =**2**
OUT:	1 14 21 25 30 43 47 =**7**

DEPROTECTION	DATE____/___/____	CHECK	MADE BY	REVIEWED BY
4MP 20% TritonX100 1%/DMF(2x10')		|_____|___|	________________	________________
Wash DMF(3x1')		|__|__|__|	________________	________________
Wash IPA(1x1')		|_________|	________________	________________
Wash BPB 1%/DMF(1x2')		|_________|	________________	________________
Wash DMF(2x1')		|_____|___|	________________	________________
Wash DCM(1x1')		|_________|	________________	________________

Coupling Cycle	DATE	HOUR	MADE BY	REVIEWED BY
AA+HBTU+OXYMA+DIPEA<br>Single (5:5:5:7,5)	|___/___/___|	__:__|__:__|	________________	________________
AA+HCTU+OXYMA+DIPEA<br>Double (5:5:5:7,5)	|___/___/___|	__:__|__:__|	________________	________________
AA+DIC+OXYMA<br>Triple (5:5:5)	|___/___/___|	__:__|__:__|	________________	________________

After coupling cycle, before checking the BPB test, wash DMF(2x1')

1:OUT	17:ARG	32:LEU	48:ILE
2:ALA	18:LYS	33:LYS	49:VAL
3:LEU	19:SER	34:TYR	50:THR
4:LEU	20:GLN	35:GLN	51:VAL
5:LEU	21:OUT	36:PHE	52:SER
6:HIS	22:VAL	37:ASN	
7:THR	23:VAL	38:LEU	
8:GLN	24:LYS	39:SER	
9:GLU	25:OUT	40:ASN	
10:LEU	26:TYR	41:THR	
12:PRO	27:VAL	42:SER	
13:LEU	28:GLN	43:OUT	
14:OUT	29:LYS	45:ILE	
15:LYS	30:OUT	46:SER	
16:ARG	31:ALA	47:OUT	

COUPLING NUMBER 12

ALA:	22 36 40 48 49 =**5**
ASP:	20 26 =**2**
GLU:	13 18 23 27 37 =**5**
PHE:	50 51 =**2**
GLY:	15 52 =**2**
HIS:	34 =**1**
ILE:	28 =**1**
LYS:	7 =**1**
LEU:	12 24 29 38 45 =**5**
MET:	6 =**1**
ASN:	17 =**1**
PRO:	2 46 =**2**
GLN:	4 5 =**2**
ARG:	10 =**1**
SER:	39 =**1**
THR:	8 33 41 =**3**
VAL:	19 32 =**2**
TYR:	3 31 35 =**3**
OUT:	9 16 42 =**3**

DEPROTECTION DATE____/___/____	CHECK	MADE BY	REVIEWED BY
4MP 20% TritonX100 1%/DMF(2x10')	|____|___|	____________	____________
Wash DMF(3x1')	|__|__|__|	____________	____________
Wash IPA(1x1')	|________|	____________	____________
Wash BPB 1%/DMF(1x2')	|________|	____________	____________
Wash DMF(2x1')	|____|___|	____________	____________
Wash DCM(1x1')	|________|	____________	____________

Coupling Cycle	DATE	HOUR	MADE BY	REVIEWED BY
AA+HBTU+OXYMA+DIPEA Single (5:5:5:7,5)	|___/___/___|	__:__|__:__|	____________	____________
AA+HCTU+OXYMA+DIPEA Double (5:5:5:7,5)	|___/___/___|	__:__|__:__|	____________	____________
AA+DIC+OXYMA Triple (5:5:5)	|___/___/___|	__:__|__:__|	____________	____________

After coupling cycle, before checking the BPB test, wash DMF(2x1')

2:PRO	19:VAL	37:GLU
3:TYR	20:ASP	38:LEU
4:GLN	22:ALA	39:SER
5:GLN	23:GLU	40:ALA
6:MET	24:LEU	41:THR
7:LYS	26:ASP	42:OUT
8:THR	27:GLU	45:LEU
9:OUT	28:ILE	46:PRO
10:ARG	29:LEU	48:ALA
12:LEU	31:TYR	49:ALA
13:GLU	32:VAL	50:PHE
15:GLY	33:THR	51:PHE
16:OUT	34:HIS	52:GLY
17:ASN	35:TYR	
18:GLU	36:ALA	

COUPLING NUMBER 13

ALA:	13 34 =**2**
ASP:	5 51 =**2**
GLU:	7 =**1**
GLY:	28 =**1**
ILE:	10 =**1**
LYS:	29 32 35 39 52 =**5**
LEU:	19 20 26 27 33 37 =**6**
ASN:	3 17 18 45 =**4**
PRO:	8 36 38 =**3**
GLN:	12 31 49 =**3**
ARG:	22 50 =**2**
SER:	2 6 40 =**3**
THR:	46 =**1**
VAL:	15 23 =**2**
TYR:	4 =**1**
OUT:	24 41 48 =**3**

DEPROTECTION	DATE____/___/____	CHECK	MADE BY	REVIEWED BY
4MP 20% TritonX100 1%/DMF(2x10')		|____|___|	________________	________________
Wash DMF(3x1')		|__|__|__|	________________	________________
Wash IPA(1x1')		|_________|	________________	________________
Wash BPB 1%/DMF(1x2')		|_________|	________________	________________
Wash DMF(2x1')		|____|___|	________________	________________
Wash DCM(1x1')		|_________|	________________	________________

Coupling Cycle	DATE	HOUR	MADE BY	REVIEWED BY
AA+HBTU+OXYMA+DIPEA Single (5:5:5:7,5)	|___/___/___|	__:__|__:__|	________________	________________
AA+HCTU+OXYMA+DIPEA Double (5:5:5:7,5)	|___/___/___|	__:__|__:__|	________________	________________
AA+DIC+OXYMA Triple (5:5:5)	|___/___/___|	__:__|__:__|	________________	________________

After coupling cycle, before checking the BPB test, wash DMF(2x1')

2:SER	22:ARG	39:LYS
3:ASN	23:VAL	40:SER
4:TYR	24:OUT	41:OUT
5:ASP	26:LEU	45:ASN
6:SER	27:LEU	46:THR
7:GLU	28:GLY	48:OUT
8:PRO	29:LYS	49:GLN
10:ILE	31:GLN	50:ARG
12:GLN	32:LYS	51:ASP
13:ALA	33:LEU	52:LYS
15:VAL	34:ALA	
17:ASN	35:LYS	
18:ASN	36:PRO	
19:LEU	37:LEU	
20:LEU	38:PRO	

COUPLING NUMBER 14

ALA: 15 34 =**2**
ASP: 19 33 =**2**
GLU: 13 =**1**
HIS: 32 =**1**
ILE: 12 =**1**
LYS: 22 27 28 31 37 =**5**
LEU: 10 18 38 =**3**
PRO: 36 =**1**
GLN: 4 26 =**2**
ARG: 5 51 =**2**
SER: 8 23 50 =**3**
THR: 2 3 =**2**
VAL: 20 39 40 =**3**
OUT: 6 7 17 29 35 45 46 49 52 =**9**

DEPROTECTION DATE____/___/____	CHECK	MADE BY	REVIEWED BY
4MP 20% TritonX100 1%/DMF(2x10')	|____|___|	________________	________________
Wash DMF(3x1')	|__|__|__|	________________	________________
Wash IPA(1x1')	|_________|	________________	________________
Wash BPB 1%/DMF(1x2')	|_________|	________________	________________
Wash DMF(2x1')	|____|___|	________________	________________
Wash DCM(1x1')	|_________|	________________	________________

Coupling Cycle	DATE	HOUR	MADE BY	REVIEWED BY
AA+HBTU+OXYMA+DIPEA Single (5:5:5:7,5)	|___/___/___|	|__:__|__:__|	________________	________________
AA+HCTU+OXYMA+DIPEA Double (5:5:5:7,5)	|___/___/___|	|__:__|__:__|	________________	________________
AA+DIC+OXYMA Triple (5:5:5)	|___/___/___|	|__:__|__:__|	________________	________________

After coupling cycle, before checking the BPB test, wash DMF(2x1')

2:THR	22:LYS	40:VAL
3:THR	23:SER	45:OUT
4:GLN	26:GLN	46:OUT
5:ARG	27:LYS	49:OUT
6:OUT	28:LYS	50:SER
7:OUT	29:OUT	51:ARG
8:SER	31:LYS	52:OUT
10:LEU	32:HIS	
12:ILE	33:ASP	
13:GLU	34:ALA	
15:ALA	35:OUT	
17:OUT	36:PRO	
18:LEU	37:LYS	
19:ASP	38:LEU	
20:VAL	39:VAL	

COUPLING NUMBER 15

ALA: 27 51 =**2**
ASP: 20 =**1**
GLU: 28 =**1**
PHE: 33 37 =**2**
GLY: 38 =**1**
HIS: 31 =**1**
ILE: 3 =**1**
LYS: 50 =**1**
LEU: 8 34 40 =**3**
PRO: 39 =**1**
GLN: 32 =**1**
ARG: 19 22 =**2**
SER: 2 10 12 23 36 =**5**
VAL: 4 =**1**
OUT: 5 13 15 18 26 =**5**

DEPROTECTION DATE____/___/____	CHECK	MADE BY	REVIEWED BY
4MP 20% TritonX100 1%/DMF(2x10')	|_____|___|	________________	________________
Wash DMF(3x1')	|__|__|__|	________________	________________
Wash IPA(1x1')	|_________|	________________	________________
Wash BPB 1%/DMF(1x2')	|_________|	________________	________________
Wash DMF(2x1')	|_____|___|	________________	________________
Wash DCM(1x1')	|_________|	________________	________________

Coupling Cycle	DATE	HOUR	MADE BY	REVIEWED BY
AA+HBTU+OXYMA+DIPEA Single (5:5:5:7,5)	|___/___/___|	|__:__|__:__|	________________	________________
AA+HCTU+OXYMA+DIPEA Double (5:5:5:7,5)	|___/___/___|	|__:__|__:__|	________________	________________
AA+DIC+OXYMA Triple (5:5:5)	|___/___/___|	|__:__|__:__|	________________	________________

After coupling cycle, before checking the BPB test, wash DMF(2x1')

2:SER	27:ALA
3:ILE	28:GLU
4:VAL	31:HIS
5:OUT	32:GLN
8:LEU	33:PHE
10:SER	34:LEU
12:SER	36:SER
13:OUT	37:PHE
15:OUT	38:GLY
18:OUT	39:PRO
19:ARG	40:LEU
20:ASP	50:LYS
22:ARG	51:ALA
23:SER	
26:OUT	

COUPLING NUMBER 16

ASP: 23 =**1**
GLU: 20 =**1**
GLY: 40 =**1**
ILE: 28 =**1**
LYS: 4 22 =**2**
LEU: 19 =**1**
ASN: 34 =**1**
PRO: 12 33 36 =**3**
GLN: 32 =**1**
ARG: 2 =**1**
SER: 8 =**1**
THR: 39 =**1**
VAL: 31 38 =**2**
TYR: 37 =**1**
OUT: 3 10 27 50 51 =**5**

DEPROTECTION DATE____/___/____	CHECK	MADE BY	REVIEWED BY
4MP 20% TritonX100 1%/DMF(2x10')	|____|___|	__________	__________
Wash DMF(3x1')	|__|__|__|	__________	__________
Wash IPA(1x1')	|________|	__________	__________
Wash BPB 1%/DMF(1x2')	|________|	__________	__________
Wash DMF(2x1')	|____|___|	__________	__________
Wash DCM(1x1')	|________|	__________	__________

Coupling Cycle	DATE	HOUR	MADE BY	REVIEWED BY
AA+HBTU+OXYMA+DIPEA<br>Single (5:5:5:7,5)	|___/___/___|	__:__|__:__|	__________	__________
AA+HCTU+OXYMA+DIPEA<br>Double (5:5:5:7,5)	|___/___/___|	__:__|__:__|	__________	__________
AA+DIC+OXYMA<br>Triple (5:5:5)	|___/___/___|	__:__|__:__|	__________	__________

After coupling cycle, before checking the BPB test, wash DMF(2x1')

2:ARG	34:ASN
3:OUT	36:PRO
4:LYS	37:TYR
8:SER	38:VAL
10:OUT	39:THR
12:PRO	40:GLY
19:LEU	50:OUT
20:GLU	51:OUT
22:LYS	
23:ASP	
27:OUT	
28:ILE	
31:VAL	
32:GLN	
33:PRO	

COUPLING NUMBER 17

ASP: 31 =**1**
LEU: 22 34 =**2**
ASN: 20 =**1**
PRO: 40 =**1**
ARG: 8 36 =**2**
SER: 4 19 23 38 =**4**
THR: 33 =**1**
TRP: 2 =**1**
TYR: 12 =**1**
OUT: 28 32 37 39 =**4**

DEPROTECTION DATE____/___/____ CHECK MADE BY REVIEWED BY

4MP 20% TritonX100 1%/DMF(2x10') |____|___|________________ ________________

Wash DMF(3x1') |__|__|__|________________ ________________

Wash IPA(1x1') |_________|________________ ________________

Wash BPB 1%/DMF(1x2') |_________|________________ ________________

Wash DMF(2x1') |____|___|________________ ________________

Wash DCM(1x1') |_________|________________ ________________

Coupling Cycle DATE HOUR MADE BY REVIEWED BY

AA+HBTU+OXYMA+DIPEA
Single (5:5:5:7,5) |___/___/___|__:__|__:__|________________ ________________

AA+HCTU+OXYMA+DIPEA
Double (5:5:5:7,5) |___/___/___|__:__|__:__|________________ ________________

AA+DIC+OXYMA
Triple (5:5:5) |___/___/___|__:__|__:__|________________ ________________

After coupling cycle, before checking the BPB test, wash DMF(2x1')

2:TRP 38:SER
4:SER 39:OUT
8:ARG 40:PRO
12:TYR
19:SER
20:ASN
22:LEU
23:SER
28:OUT
31:ASP
32:OUT
33:THR
34:LEU
36:ARG
37:OUT

COUPLING NUMBER 18

ASP: 40 =**1**
PHE: 12 =**1**
GLY: 34 38 =**2**
GLN: 8 =**1**
SER: 4 =**1**
THR: 31 =**1**
VAL: 2 =**1**
OUT: 19 20 22 23 33 36 =**6**

DEPROTECTION	DATE____/___/____	CHECK	MADE BY	REVIEWED BY
4MP 20% TritonX100 1%/DMF(2x10')		|_____|___|	________________	________________
Wash DMF(3x1')		|__|__|__|	________________	________________
Wash IPA(1x1')		|_________|	________________	________________
Wash BPB 1%/DMF(1x2')		|_________|	________________	________________
Wash DMF(2x1')		|_____|___|	________________	________________
Wash DCM(1x1')		|_________|	________________	________________

Coupling Cycle	DATE	HOUR	MADE BY	REVIEWED BY
AA+HBTU+OXYMA+DIPEA<br>Single (5:5:5:7,5)	|___/___/___|	__:__|__:__|	________________	________________
AA+HCTU+OXYMA+DIPEA<br>Double (5:5:5:7,5)	|___/___/___|	__:__|__:__|	________________	________________
AA+DIC+OXYMA<br>Triple (5:5:5)	|___/___/___|	__:__|__:__|	________________	________________

After coupling cycle, before checking the BPB test, wash DMF(2x1')

2:VAL
4:SER
8:GLN
12:PHE
19:OUT
20:OUT
22:OUT
23:OUT
31:THR
33:OUT
34:GLY
36:OUT
38:GLY
40:ASP

COUPLING NUMBER 19

PHE: 4 =**1**
LEU: 8 =**1**
PRO: 31 =**1**
OUT: 2 12 34 38 40 =**5**

DEPROTECTION	DATE____/___/____	CHECK	MADE BY	REVIEWED BY
4MP 20% TritonX100 1%/DMF(2x10')		|____|___|	________________	________________
Wash DMF(3x1')		|__|__|__|	________________	________________
Wash IPA(1x1')		|_________|	________________	________________
Wash BPB 1%/DMF(1x2')		|_________|	________________	________________
Wash DMF(2x1')		|____|___|	________________	________________
Wash DCM(1x1')		|_________|	________________	________________

Coupling Cycle	DATE	HOUR	MADE BY	REVIEWED BY
AA+HBTU+OXYMA+DIPEA Single (5:5:5:7,5)	|___/___/___|	__:__|__:__|	________________	________________
AA+HCTU+OXYMA+DIPEA Double (5:5:5:7,5)	|___/___/___|	__:__|__:__|	________________	________________
AA+DIC+OXYMA Triple (5:5:5)	|___/___/___|	__:__|__:__|	________________	________________

After coupling cycle, before checking the BPB test, wash DMF(2x1')

2:OUT
4:PHE
8:LEU
12:OUT
31:PRO
34:OUT
38:OUT
40:OUT

COUPLING NUMBER 20

SER: 31 =**1**
THR: 4 =**1**
OUT: 8 =**1**

DEPROTECTION	DATE____/___/____	CHECK	MADE BY	REVIEWED BY
4MP 20% TritonX100 1%/DMF(2x10')		|____|___|	____________	____________
Wash DMF(3x1')		|__|__|__|	____________	____________
Wash IPA(1x1')		|________|	____________	____________
Wash BPB 1%/DMF(1x2')		|________|	____________	____________
Wash DMF(2x1')		|____|___|	____________	____________
Wash DCM(1x1')		|________|	____________	____________

Coupling Cycle	DATE	HOUR	MADE BY	REVIEWED BY
AA+HBTU+OXYMA+DIPEA<br>Single (5:5:5:7,5)	|___/___/___|	__:__|__:__|	____________	____________
AA+HCTU+OXYMA+DIPEA<br>Double (5:5:5:7,5)	|___/___/___|	__:__|__:__|	____________	____________
AA+DIC+OXYMA<br>Triple (5:5:5)	|___/___/___|	__:__|__:__|	____________	____________

After coupling cycle, before checking the BPB test, wash DMF(2x1')

4:THR
8:OUT
31:SER

COUPLING NUMBER 21

OUT: 4 31 =**2**

DEPROTECTION	DATE____/___/____	CHECK	MADE BY	REVIEWED BY
4MP 20% TritonX100 1%/DMF(2x10')		|____|___|	______________	______________
Wash DMF(3x1')		|__|__|__|	______________	______________
Wash IPA(1x1')		|________|	______________	______________
Wash BPB 1%/DMF(1x2')		|________|	______________	______________
Wash DMF(2x1')		|____|___|	______________	______________
Wash DCM(1x1')		|________|	______________	______________

Coupling Cycle	DATE	HOUR	MADE BY	REVIEWED BY
AA+HBTU+OXYMA+DIPEA<br>Single (5:5:5:7,5)	|___/___/___|	__:__|__:__|	______________	______________
AA+HCTU+OXYMA+DIPEA<br>Double (5:5:5:7,5)	|___/___/___|	__:__|__:__|	______________	______________
AA+DIC+OXYMA<br>Triple (5:5:5)	|___/___/___|	__:__|__:__|	______________	______________

After coupling cycle, before checking the BPB test, wash DMF(2x1')

4:OUT
31:OUT
